# The Epithelial-Mesenchymal Transition Factor SNAIL Paradoxically Enhances Reprogramming

**DOI:** 10.1016/j.stemcr.2014.09.008

**Published:** 2014-10-11

**Authors:** Juli J. Unternaehrer, Rui Zhao, Kitai Kim, Marcella Cesana, John T. Powers, Sutheera Ratanasirintrawoot, Tamer Onder, Tsukasa Shibue, Robert A. Weinberg, George Q. Daley

**Affiliations:** 1 Division of Pediatric Hematology/Oncology, Stem Cell Transplantation Program, Manton Center for Orphan Disease Research, Howard Hughes Medical Institute, Children’s Hospital Boston and Dana Farber Cancer Institute, Harvard University, Cambridge, MA 02138, USA; 2 Department of Biological Chemistry and Molecular Pharmacology, Harvard Medical School, Harvard University, Cambridge, MA 02138, USA; 3 Harvard Stem Cell Institute, Harvard University, Cambridge, MA 02138, USA; 4 Whitehead Institute for Biomedical Research, 9 Cambridge Center, Cambridge, MA 02142, USA; 5 Ludwig Center for Molecular Oncology, Massachusetts Institute of Technology, 77 Massachusetts Avenue, Cambridge, MA 02139, USA; 6 Department of Biology, Massachusetts Institute of Technology, 77 Massachusetts Avenue, Cambridge, MA 02139, USA

## Abstract

Reprogramming of fibroblasts to induced pluripotent stem cells (iPSCs) entails a mesenchymal to epithelial transition (MET). While attempting to dissect the mechanism of MET during reprogramming, we observed that knockdown (KD) of the epithelial-to-mesenchymal transition (EMT) factor *SNAI1 (SNAIL)* paradoxically reduced, while overexpression enhanced, reprogramming efficiency in human cells and in mouse cells, depending on strain. We observed nuclear localization of SNAI1 at an early stage of fibroblast reprogramming and using mouse fibroblasts expressing a knockin *SNAI1*-YFP reporter found cells expressing SNAI1 reprogrammed at higher efficiency. We further demonstrated that SNAI1 binds the let-7 promoter, which may play a role in reduced expression of let-7 microRNAs, enforced expression of which, early in the reprogramming process, compromises efficiency. Our data reveal an unexpected role for the EMT factor SNAI1 in reprogramming somatic cells to pluripotency.

## Highlights

• Knockdown of SNAIL reduces and overexpression enhances reprogramming
• SNAIL-YFP-positive fractions reprogram at higher efficiency
• Let-7 decreases early in reprogramming, and expression of SNAIL reduces let-7
• SNAIL binds to the promoters of let-7 family members during reprogramming

## Teaser

Unternaehrer and colleagues show that SNAI1, a factor associated with epithelial-to-mesenchymal transition, promotes reprogramming of somatic cells to pluripotency, a counterintuitive discovery since reprogramming requires the opposite process, mesenchymal-to-epithelial transition. SNAI1 expression is temporally associated with downregulation of the microRNA let-7, and SNAI1 binds the let-7 promoter consistent with direct regulation.

## Introduction

Reprogramming somatic cells to induced pluripotent stem cells (iPSCs) holds great promise for disease modeling and therapeutic applications. Among the challenges that remain is the extended time frame and variable efficiency of transcription factor-based reprogramming; in most cases, fewer than 0.05% of the target population will form bona fide iPSC colonies (Chan et al., 2009; Takahashi et al., 2007). Improving our knowledge of the mechanisms of reprogramming should facilitate more efficient reprogramming.

Studies aimed at elucidating the mechanism of reprogramming somatic cells to pluripotency have revealed a multistep process in which exogenous OCT4, SOX2, KLF4, and MYC (OSKM) expression initiates events resulting in endogenous expression of pluripotency factors and a stable iPSC phenotype. Fibroblasts, the somatic cells most often targeted for reprogramming, are quintessential mesenchymal cells, whereas embryonic stem (ES) or iPSCs express epithelial markers. Completion of the prolonged reprogramming process entails upregulation of epithelial factors and downregulation of mesenchymal factors—a classic mesenchymal to epithelial transition (MET) (Samavarchi-Tehrani et al., 2010). KLF4 has been shown to induce E-cadherin and other epithelial factors, whereas SOX2 and OCT4 downregulate SNAI1 (Li et al., 2010). Thus, a mechanistic link between MET and fibroblast reprogramming has been proposed.

However, considerable data suggest that reprogramming is not a simple MET from start to finish. A sequential application of reprogramming factors, shown to induce an early epithelial-to-mesenchymal transition (EMT), improves efficiency, as does early transforming growth factor-β (TGF-β) treatment or Slug expression (Liu et al., 2013). Wnt signaling, which promotes EMT, enhances reprogramming if activated early in the process (Marson et al., 2008). Inhibitors of TGF-β signaling, which promote MET, can enhance reprogramming if administered concomitant with OSKM expression (Ichida et al., 2009) but antagonize reprogramming if administered in the 3 preceding days (Maherali and Hochedlinger, 2009). Taken together, these data imply that induction of mesenchymal fates may play a positive role in the reprogramming process. The role of EMT factors in induction of pluripotency has not been extensively explored.

The transcription factor SNAI1 represses epithelial factors such as E-cadherin, and its expression is sufficient for EMT (Cano et al., 2000). It exerts a positive influence on the expression of stemness factors, including SOX2 and KLF4, via effects on microRNAs (miRs) (Garibaldi et al., 2012), and has been shown to decrease proliferation while preventing apoptosis (Vega et al., 2004).

LIN28, a regulator of miR biogenesis and an alternative reprogramming factor (Viswanathan et al., 2008; Yu et al., 2007), inhibits the processing and maturation of let-7 and is in turn a let-7 target (Rybak et al., 2008). Mature let-7 family miRs, regulators of developmental timing (Ambros, 2011), are absent in pluripotent cells and are expressed at high levels in differentiated cell populations (Viswanathan et al., 2008). Let-7 inhibits expression of pluripotency factors (including LIN28, c-MYC, and SALL4) (Melton et al., 2010) and cell cycle regulators critical for the ES cell phenotype (such as CDK6, CDC25A, and cyclin D) (reviewed in (Mallanna and Rizzino, 2010). Expression of let-7 miRs can promote differentiation of pluripotent stem cells, and a let-7 inhibitor promotes dedifferentiation; thus, let-7 downregulation is likely essential to reprogramming (Melton et al., 2010). Let-7 inhibition stimulates OSK reprogramming efficiency (without c-MYC) to the same extent as does c-MYC, and forced let-7 expression decreases reprogramming efficiency (Worringer et al., 2014). Moreover, a connection between EMT factors and the transcriptional regulation of let-7 has been reported (Chang et al., 2011; Kong et al., 2010; Li et al., 2009).

Here we set out to evaluate the roles of transcriptional regulators associated with EMT in iPSC reprogramming. We were intrigued by data from published reports that EMT factors (including *SNAI1*) increased during the early stages of reprogramming (Mikkelsen et al., 2008; Samavarchi-Tehrani et al., 2010). We were likewise perplexed by the continued expression of this and other EMT factors at time points when genes downregulated by them were strongly expressed. Thus, we examined the roles of SNAI1 during reprogramming.

## Results

### Effects of SNAI1 on Reprogramming

We investigated expression levels of *SNAI1* and pluripotency markers *POU5F1* (OCT4) and *LIN28A* during OSKM reprogramming (Figures S1A and S1B available online) of fibroblasts and keratinocytes and found that *SNAI1* expression was variably increased through intermediate stages in reprogramming in both mouse and human cultures. Our expression data were consistent with published microarrays showing, in fibroblasts, upregulation of *SNAI1* and other EMT factors early in mouse reprogramming, while reduced *SNAI1* levels were not observed until day 8 (Mikkelsen et al., 2008; Samavarchi-Tehrani et al., 2010). Although the kinetics of reprogramming are variable between experiments, we consistently observed early upregulation of *SNAI1*, leading us to hypothesize that it might play a positive role.

**Figure 1:**
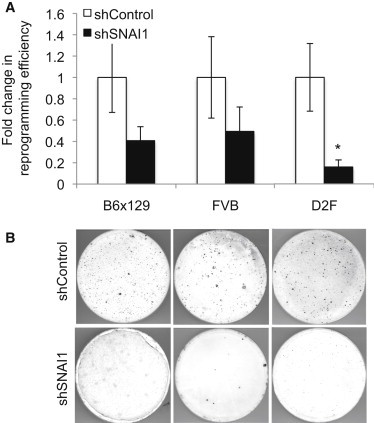
KD of *SNAI1* in Fibroblasts Decreases Reprogramming Efficiency (A) In MEFs or D2F, *SNAI1* was knocked down by three different shRNAs individually, followed by retroviral reprogramming or dox addition, and colonies were counted based on morphology and SSEA-1 or Tra1-60 labeling after 21 or 28 days, respectively, normalized to cell number on day 3. Shown is fold change relative to control (scrambled shRNA); bars indicate shControl (white) and shSNAI1 (black). n = 10–22 in four to nine biological replicates. Error bars show SEM. ^∗^p < 0.05. B6×129: p = 0.06, FVB: p = 0.08. See also Figure S1. (B) SSEA-1 labeling of B6×129 (left), FVB (middle), or Tra1-60 labeling of D2F (right); control shRNA (upper) and *SNAI1* KD (lower) are shown.

To explore the role of *SNAI1*, we knocked it down in murine fibroblasts and evaluated the effects on the efficiency of reprogramming. We confirmed that *SNAI1* was knocked down by quantitative PCR (qPCR) and immunoblot (Figures S1C and S1D). Unexpectedly, we observed that knockdown (KD) caused a trend toward decreased rather than increased reprogramming efficiency in two different mouse strains, C57BL/6 × 129 (B6×129) and mouse strain Friend Virus B (FVB) (Figures 1A and 1B). To enable analysis of live colonies, we used colony morphology and stage-specific embryonic antigen 1 (SSEA-1) expression as our indicator of successful reprogramming, after showing that numbers based on Nanog expression in fixed colonies correlated with those obtained by SSEA-1 labeling (Figure S1E). To investigate whether EMT factors play similar roles in reprogramming of human somatic cells, we employed a “secondary” reprogramming system in which fibroblasts were differentiated from iPSCs carrying doxycycline (dox)-inducible reprogramming factors (Figure S2A) (Kim et al., 2011). In secondary human fibroblasts (D2F), KD of *SNAI1* likewise compromised reprogramming (Figures 1A, 1B, and S1C).

**Figure 2:**
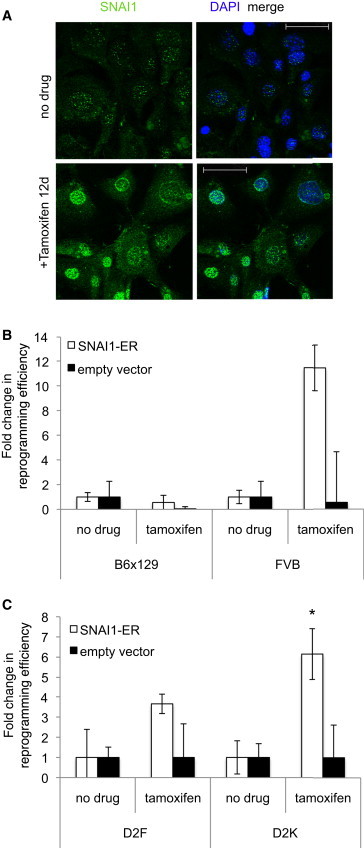
Overexpression of SNAI1 via ER Fusion Improves Reprogramming Efficiency (A) A B6×129 line expressing SNAI1-ER was created by retroviral transduction. Cells were treated with TMX for 12 days and fixed and labeled. With (lower) or without induction (upper); SNAI1 labeling, green; DAPI, blue; 40×, scale bar represents 50 μm. (B) Mouse fibroblasts of indicated strain were reprogrammed and efficiency calculated based on colony morphology and SSEA-1 labeling. (C) D2F or D2K were reprogrammed as in B and Tra 1-60+ colonies labeled. (B and C) White bars, SNAI1-ER; black, empty vector; fold increase over no TMX is shown. n = 3–14, 2–6 biological replicates. Error bars in (B) and (C) show SEM. ^∗^p < 0.05. See also Figure S2.

We then enforced expression of SNAI1 by tamoxifen (TMX) induction of an estrogen receptor (ER) fusion construct prior to the initiation of reprogramming (Mani et al., 2008). In fibroblasts, levels of expression of EMT factors are relatively low, and they increase upon TGF-β treatment (Figure S2B). While constitutive SNAI1 overexpression during reprogramming has been shown to decrease efficiency (Li et al., 2010), its effects during the early timeframe have not been tested. We monitored rates of proliferation since SNAI1 is known to play a role in cell cycle regulation (Vega et al., 2004). Upon TMX addition to control versus SNAI1-expressing cells, changes in proliferation rates were not significant (Figure S2C). SNAI1 expression in the nucleus increased upon TMX treatment (Figures 2A and S2D). Cells in which SNAI1 had been induced were then reprogrammed by viral transduction following cessation of TMX. In MEFs from FVB mice, overexpression of SNAI1 prior to reprogramming increased efficiency, while in B6×129, a strain with high baseline reprogramming efficiency, SNAI1 did not further augment reprogramming (Figures 2B and S2H). *SNAI1* expression in the two strains was similar (Figure S2I).

We reasoned that if SNAI1 plays a role in reprogramming we should observe a more dramatic effect on the reprogramming of epithelial cells, such as keratinocytes, because their intrinsic level of expression of *SNAI1*, although not absent, is lower than in fibroblasts (Figure S2F). To test this and to discern the effect in human cells, we overexpressed SNAI1 via ER fusion in human fibroblasts and keratinocytes prior to reprogramming and confirmed TMX-induced nuclear translocation (Figure S2E). Overexpression of SNAI1 in both cell types caused an enhancement of colony formation, with a more pronounced effect in epithelial cells (Figure 2C), confirming that both mesenchymal and epithelial cell types reprogram more efficiently when SNAI1 is expressed. Accordingly, with respect to fibroblasts, SNAI1 expression is more markedly upregulated in keratinocytes, presumably due to lower starting levels (Figures S1A and S1B).

### Cells Expressing Endogenous SNAI1 Reprogram More Efficiently

**Figure 3:**
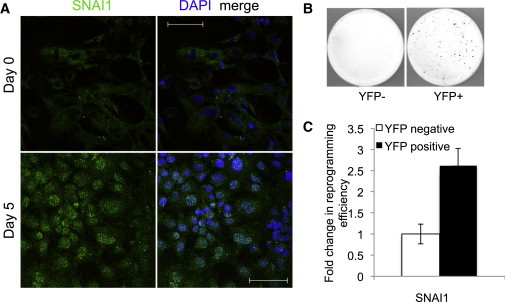
SNAI1 Expression, Upregulated Early in Reprogramming, Predicts Higher Reprogramming Efficiency (A) On days 0 and 5 of reprogramming, B6×129 fibroblasts were labeled with anti-SNAI1 (green, left panels) and DAPI (blue, overlaid with green in right panels); 40×, scale bar represents 50 μm. (B) YFP-positive and -negative sorted MEFs from *SNAI1*-YFP knockin mice were retrovirally reprogrammed. SSEA1+ colonies were counted on day 21; representative images are shown. (C) Quantification of colonies, normalized for cell number on day 3. n = 7–19, 5 biological replicates. Error bars show SEM. See also Figure S3.

Before reprogramming, MEFs expressed low levels of *SNAI1* (Figure 3A, upper panel). Early in the reprogramming process (days 1–7), endogenous SNAI1 became localized to the nucleus (Figure 3A, lower panel; day 5 shown; Figure S3D).

To test whether cells expressing endogenous SNAI1 are more efficiently reprogrammed, we isolated cells from mice with a knockin reporter construct that enables selection for cells expressing SNAI1 by virtue of yellow fluorescent protein coexpression from an internal ribosomal entry site (T.S. and R.A.W., unpublished data). We noted varying proportions of YFP-positive cells in tail tip fibroblasts and mouse embryonic fibroblasts (MEFs). Sorted *SNAI1*-YFP-positive and -negative populations expressed higher and lower levels of *SNAI1* mRNA, respectively (Figure S3A). YFP-positive fractions showed an increased reprogramming efficiency for SNAI1 (6.5×) as compared with negative populations (Figure 3B). We found that upon culture fewer YFP-negative than YFP-positive cells resulted, either because of proliferative or cell death differences, but after normalizing for cell number differences (as described in Experimental Procedures), enhancement was still seen for SNAI1 (2.6×, Figure 3C).

We observed an increase in *SNAI1* expression early in reprogramming in B6×129, but not in FVB (Figure S3B), the strain in which SNAI1 overexpression increased efficiency. *SNAI1* expression was also seen in mouse peripheral blood reprogramming (Figure S3C). Thus, an increase in *SNAI1* expression was seen across mesenchymal, epithelial, and peripheral blood cell types and could be observed by monitoring RNA or protein.

### *SNAI1* Expression Is Temporally Associated with Let-7 Downregulation

**Figure 4:**
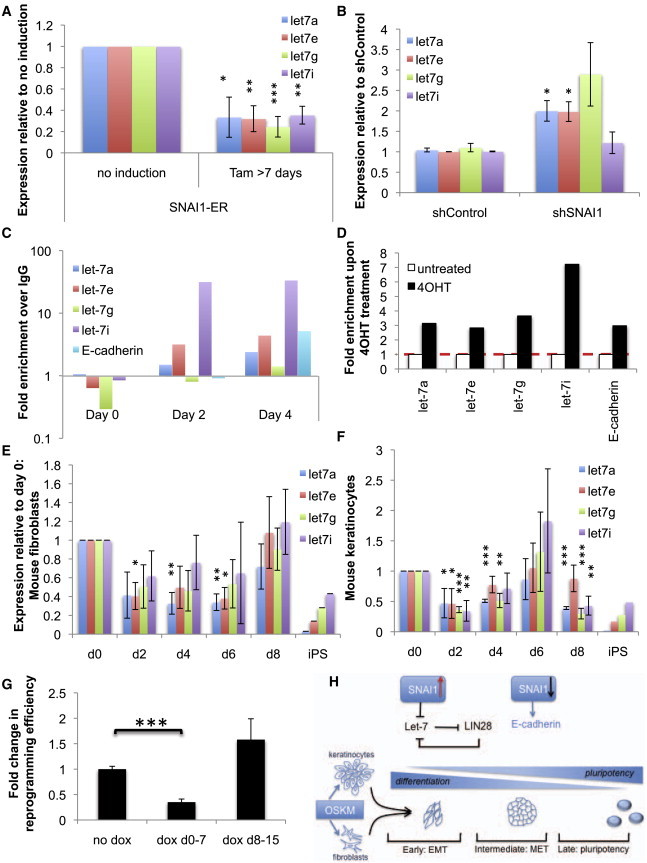
Snail Expression Is Temporally Associated with Downregulation of Let-7 (A) qPCR analyses on let-7 expression upon SNAI1 overexpression. After 7 or 10 days of TMX treatment, B6×129 fibroblasts stably expressing SNAI1-ER were tested for levels of expression of let-7 revealed by TaqMan qPCR (n = 3–4 biological replicates). Error bars show SEM. (B) qPCR analyses of let-7 expression upon *SNAI1* KD. B6×129 fibroblasts with lentiviral *SNAI1* KD were analyzed for expression of let-7 (n = 3 biological replicates). Error bars show SEM. (C) ChIP analysis of endogenous SNAI1 on promoters of let-7 genes. Samples from iOSKM MEF reprogramming were harvested at days 0, 2, and 4, prepared for ChIP, and analyzed for SNAI1 binding to E boxes in the promoters of let-7a, let-7e, let-7g, and let-7i. Binding to the E-cadherin promoter functioned as a positive control. IgG, control antibody. n = 3 biological replicates; representative experiment shown. (D) ChIP analysis of SNAI1-ER upon TMX treatment. iOSKM MEFs stably expressing SNAI1-ER (see Figure 2) were harvested before or after 10 days of induction with TMX. Binding to the promoters of let-7a, let-7e, let-7g, and let-7i was analyzed by ChIP. White bars, untreated; black bars, + TMX. (E and F) qPCR analyses of let-7 expression during the reprogramming of iOSKM (B6×129) fibroblasts and keratinocytes. RNA samples isolated from days 0, 2, 4, 6, and 8 of fibroblast (E, n = 4–5) or keratinocyte (F, n = 3 biological replicates) reprogramming was analyzed by TaqMan qPCR for let-7. ^∗^p < 0.05; ^∗∗^p < 0.01; ^∗∗∗^p < 0.001. Error bars show SEM. See also Figure S4. (G) Forced expression of let-7 in reprogramming. iLet-7 MEFs were retrovirally reprogrammed with or without dox addition at days 0–7 and day 8–15. n = 3–5, 3 biological replicates. Error bars show SEM. ^∗∗∗^p < 0.001. (H) Schematic representation of hypothesis. After expression of four pluripotency factors, *SNAI1* upregulation leads to let-7 downregulation. Targeting of LIN28 and other pluripotency factors is thus removed, and the differentiated state is destabilized, allowing expression of pluripotency factors.

Next, we explored the potential mechanism by which SNAI1 enhances reprogramming, noting the references that link EMT with downregulation of the let-7 family of tumor suppressor miRs (Chang et al., 2011; Kong et al., 2010; Li et al., 2009; Yang et al., 2012). Using inducible Snail ER, we observed downregulation of let-7 after 7 days of TMX treatment in mouse fibroblasts (Figures 4A and S4A). KD of *SNAI1* resulted in increased let-7 expression (Figure 4B). Chromatin immunoprecipitation (ChIP) confirmed that SNAI1 binds the promoters of several let-7 family members during early stages of reprogramming in B6×129 fibroblasts (Figure 4C) and in FVB overexpressing SNAI1-ER more so than without induction (Figure S4B). Upon TMX treatment of SNAI1-ER expressing fibroblasts (without reprogramming), SNAI1 binding to let-7 members similarly increases (Figure 4D).

We evaluated expression of let-7 during OSKM-induced reprogramming and found let-7a, let-7e, let-7g, and let-7i decreased in both fibroblasts and keratinocytes in the early phase (Figures 4E, 4F, S4C (parallel fibroblast data for Figure S1A), and S4D). A similar trend can be seen in the case of mouse peripheral blood (Figure S4E). To understand the role of let-7 in reprogramming, we expressed let-7 in MEFs from a strain of mice carrying a dox-inducible transgene at various stages of reprogramming (Zhu et al., 2011). We found that let-7 overexpression compromised efficiency when done during the first, but not the second, 7 days of reprogramming (Figure 4G). We also noted a trend toward higher expression of several let-7 members in FVB than in B6×129 strain prior to reprogramming, correlating high expression with augmentation of reprogramming efficiency upon SNAI1 overexpression (Figure S4F). These data suggest downregulation of the let-7 miRs as a possible mechanism by which SNAI1 influences reprogramming (diagrammed in Figures 4H, S4G, and S4H).

## Discussion

Fibroblasts are the typical starting population for somatic cell reprogramming, and prior studies have indicated that reprogramming involves an MET. Paradoxically, however, transcription factors associated with EMT are expressed early in the reprogramming process and are not downregulated until the later stages (Samavarchi-Tehrani et al., 2010). While previously it was reported that keratinocytes could be reprogrammed with higher efficiency because of their pre-existing epithelial status (Maherali et al., 2008), a side-by-side comparison between cell types has not been done. In a secondary reprogramming system that enables direct comparison, we found mouse keratinocytes were reprogrammed less efficiently (0.02%) than fibroblasts (0.3%) (Figure S3E), and we have discovered ectopic expression of the EMT factor SNAI1 during early stages of reprogramming enhances efficiency in keratinocytes, an epithelial cell type. Thus, the effect of expression of EMT factors in the initial phase of reprogramming is not limited to mesenchymal target cell populations, but also occurs in epithelial cells, suggesting mesenchymal factor expression is an important aspect of reprogramming independent of starting cell type. Manipulating SNAI1 has led us to a multistep model of reprogramming whereby mesenchymal factors are expressed early and contribute to the reprogramming-amenable state (Koche et al., 2011), and only thereafter are pluripotency factors expressed en route to the pluripotent state seen in iPSCs (Figure 4H). Our data are in agreement with a recent study (Liu et al., 2013), which reported early enhancement of the mesenchymal state increased reprogramming efficiency.

A role for let-7 in reprogramming has been established since its inhibition increases reprogramming efficiency (Melton et al., 2010). As shown here, SNAI1 binds several let-7 promoters, and *SNAI1* expression is associated temporally with downregulation of let-7 miRs early in reprogramming, consistent with prior evidence that EMT factors suppress let-7 expression in cancer (Yang et al., 2012). Moreover, overexpressing SNAI1 in a poorly reprogramming strain augments both reprogramming efficiency and SNAI1 binding to the let-7 promoter, suggesting SNAI1 regulation of let-7 may be the basis for enhanced reprogramming efficiency. The downregulation of let-7 transcription by SNAI1 may be associated with upregulation of LIN28 by pluripotency factors, thereby potently reversing the differentiated state. While let-7 is downregulated in the first week of reprogramming, its expression appears to recover thereafter before again diminishing to near zero in the iPSC state (Figures 4E, 4F, and S4D). We have not studied this biphasic expression pattern, but we hypothesize the second wave is extinguished by LIN28.

Prior studies have demonstrated that Twist promotes a stem cell phenotype in cancer, including self-renewal (Mani et al., 2008). We hypothesize that expression of SNAI1 might similarly promote a stem cell-like phenotype in fibroblasts and keratinocytes, moving them one step closer to dedifferentiation and making them more amenable to reprogramming. We propose that suppression of let-7 miRs is a mechanism whereby SNAI1 might be acting to confer these stem cell properties.

Building on the model proposed by Samavarchi-Tehrani et al. and Li et al. demonstrating the role of MET in reprogramming, we show during the early phases of reprogramming, mesenchymal factors are expressed, and further ectopic expression of EMT factors enhances reprogramming efficiency. Our results provide a more nuanced view of the role of EMT factors in the reprogramming of both mesenchymal and epithelial cell types. Our data are corroborated by an independent study that identified *SNAI1* in an unbiased shRNA screen as a factor that enhances conversion of pre-iPS cells to a fully reprogrammed state, thereby reinforcing the conclusion that SNAI1 acts to enhance reprogramming (Gingold et al., 2014). This improved understanding of the mechanism of reprogramming will provide strategies to improve its utility for modeling and treating disease and advance our insight into the regulation of gene expression and pluripotency.

## Experimental Procedures

### Mice and Cells

All mouse studies were approved by the Boston Children’s Hospital IACUC and were done in accordance with institutional and national standards and regulations. Mouse keratinocytes were isolated from neonatal mice, cultured in CnT-07 medium (Cell-N-Tec, ZenBio) and reprogrammed at first passage. Second-generation inducible iPSCs were generated from H1 human embryonic stem cell by differentiation to fibroblasts (Park et al., 2008), transduction with inducible OSKM lentiviruses (Stadtfeld et al., 2008), and dox induction. Keratinocytes were differentiated from iPSCs by a 6 day culture in basic fibroblast growth factor-free media followed by dissociation and 3–4 day culture in keratinocyte serum-free medium (Invitrogen) +/− retronectin (Takara Shuzo) (Kim et al., 2011).

### Reprogramming

Retroviral-mediated mouse reprogramming was via pmX constructs. Second-generation inducible fibroblasts or keratinocytes were induced by addition of dox at 2 μg/ml 12–24 h after plating. In both cases, cells were replated onto irradiated MEFs (GlobalStem) 3 days (mouse) or 5 days (human) after expression of the four factors, with daily changes of mouse or human ES cell media thereafter.

### Flow Cytometry

Tail tip fibroblasts or MEFs positive or negative for YFP were sorted on a FACSAria (BD). Cells were used immediately for reprogramming or qPCR.

### ChIP Assays

ChIP analyses were carried out as described (Cesana et al., 2011).

## Supplemental Information

Document S1. Supplemental Experimental Procedures, Figures S1–S4, and Table S1

Document S2. Article plus Supplemental Information
