# Supplementary material for: The Epithelial-Mesenchymal Transition Factor SNAIL Paradoxically Enhances Reprogramming
Source: Stem Cell Reports. 2014 Oct 11;3(5):691–8. doi: 10.1016/j.stemcr.2014.09.008 (PMC4235745; doi:10.1016/j.stemcr.2014.09.008)
Supplement: Document S1. Supplemental Experimental Procedures, Figures S1–S4, and Table S1 [file mmc1.pdf]

Stem Cell Reports, Volume *3*

Supplemental Information

The Epithelial-Mesenchymal Transition Factor SNAI1 Paradoxically Enhances Reprogramming

Juli J. Unternaehrer, Rui Zhao, Kitai Kim, Marcella Cesana, John T. Powers, Sutheera Ratanasirintrawoot, Tamer Onder, Tsukasa Shibue, Robert A. Weinberg, and George Q. Daley

Inventory of Supplemental Information

Supplemental Figures and Legends:

- **Figure S1 (refers to Figure 1)**
- **Figure S2 (refers to Figure 2)**
- **Figure S3 (refers to Figure 3)**
- **Figure S4 (refers to Figure 4)**

Supplemental Table 1.

Supplemental Experimental Procedures

Supplemental References

Figure S1. Gene expression during mouse and human fibroblast and keratinocyte reprogramming.

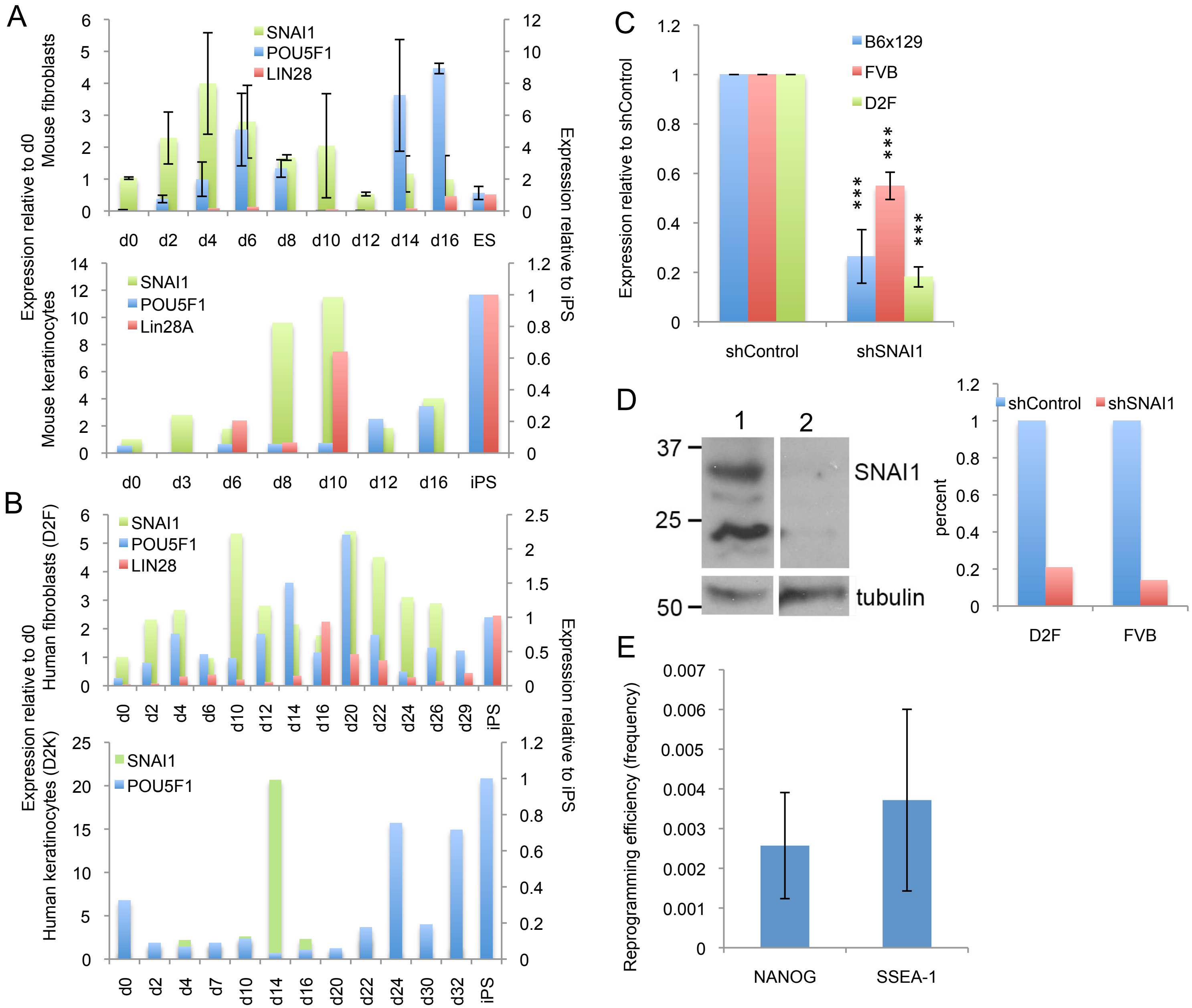

Figure S1. Time course of gene expression during mouse and human fibroblast and keratinocyte reprogramming **(Relates to Figure 1)**.

A. qPCR analyses in mouse cell reprogramming. In mouse fibroblasts (upper panel) and keratinocytes (lower panel) four factors were induced virally or by addition of dox, respectively, and samples were harvested for RNA isolation at indicated time points. After generating cDNA, samples were subjected to qRT-PCR, with normalization to GAPDH. *SNAI1* (green) relative to day 0, endogenous *POU5F1* (blue) and *LIN28* (red) relative to iPS cells. n=3-6 biological replicates; shown are 3 technical replicates for fibroblasts, representative experiment for keratinocytes.

B. qPCR analyses in human cell reprogramming. H1-derived D2F and D2K were reprogrammed by addition of dox, and samples were harvested for RNA isolation at indicated time points. After generating cDNA, samples were subjected to qRT-PCR, with normalization to Actin B. *SNAI1* (green) relative to day 0, endogenous *POU5F1* (blue) and *LIN28* (red) relative to iPS cells. n=3-4 biological replicates; representative experiment shown.

C. qPCR analyses to characterize *SNAI1* knockdown lines. After at least 7d of selection of puromycin-resistant cells, RNA was isolated and cDNA generated, and levels of expression analyzed by qRT-PCR as shown for *SNAI1*, with individual lines for each of three hairpins. Shown is average of three hairpins. n=4-6 biological replicates. ***, $p<0.001$.

D. SNAI1 expression (upper blot) in D2F expressing a control shRNA (lane 1) or shSNAI1 (lane 2) was determined by immunoblotting; lower band is a degradation product of SNAI1 (Zhou et al., 2004). Lower blot, anti-tubulin loading control. Graph at right shows knockdown efficiency in D2F and FVB relative to shControl, normalized to loading control.

E. Colony quantification by labeling with anti-SSEA-1 and anti-NANOG after fixation at day 21 of reprogramming; n=6 technical (NANOG), 2 biological with 6 technical (SSEA-1) replicates. $p>0.2$.

Figure S2. SNAI1 overexpression in human and mouse fibroblasts and keratinocytes.

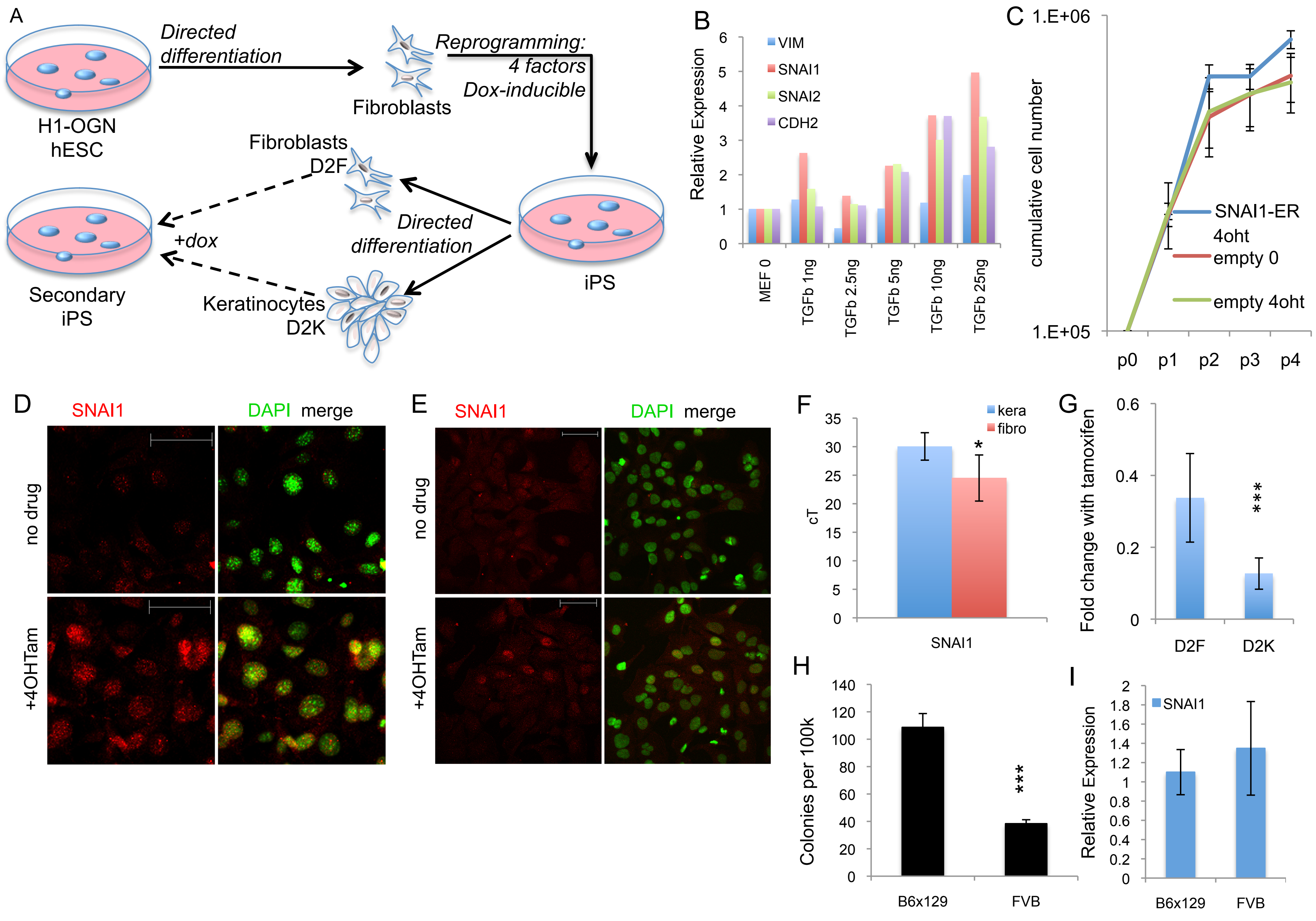

Figure S2. EMT factor overexpression in human and mouse fibroblasts and keratinocytes **(Relates to Figure 2)**.

A. H1 OGN hESCs were differentiated to fibroblasts (Park et al., 2008), followed by transduction with inducible OKSM and rtTA. Doxycycline treatment gave rise to iPS colonies which were picked and expanded. These were in turn differentiated to fibroblasts (D2F) and keratinocytes (D2K), which could then be reprogrammed by addition of Dox, giving rise to secondary iPS cells.

B. Mouse fibroblasts were treated with increasing concentrations of TGF-beta for two days, and expression of EMT factors was determined by qPCR.

C. Proliferation of fibroblasts overexpressing SNAI1-ER or empty vector, +/- tamoxifen. Cells were passaged every three days. n=3 technical replicates.

D,E. Overexpression of SNAI1 in D2F (E) by retroviral transduction and blasticidin selection, then tamoxifen treatment for 12 days, resulted in cells with nuclear-enriched SNAI1 (lower panels). Scale bar, 50um.

F. Expression of SNAI1 in keratinocytes and fibroblasts, strain B6x129. RNA from starting cell populations was processed for qPCR; shown are cT values. n=4, 3 biological replicates. *, $p<0.05$.

G. Effect of tamoxifen on human reprogramming efficiency. D2F and D2K cells bearing an empty vector plasmid, pWZL-GFP, were treated or not with 20nM tamoxifen prior to reprogramming. n=6-8, 3-4 biological replicates. ***, $p<0.001$.

H. Fibroblasts from strains B6x129 and FVB were retrovirally reprogrammed and colonies counted on day 21. n=3 biological replicates. ***, $p<0.001$.

I. SNAI1 expression in starting fibroblasts from strains B6x129 and FVB was determined by qPCR; n=3-5 biological replicates.

Figure S3. Endogenous SNAI1 expression during reprogramming

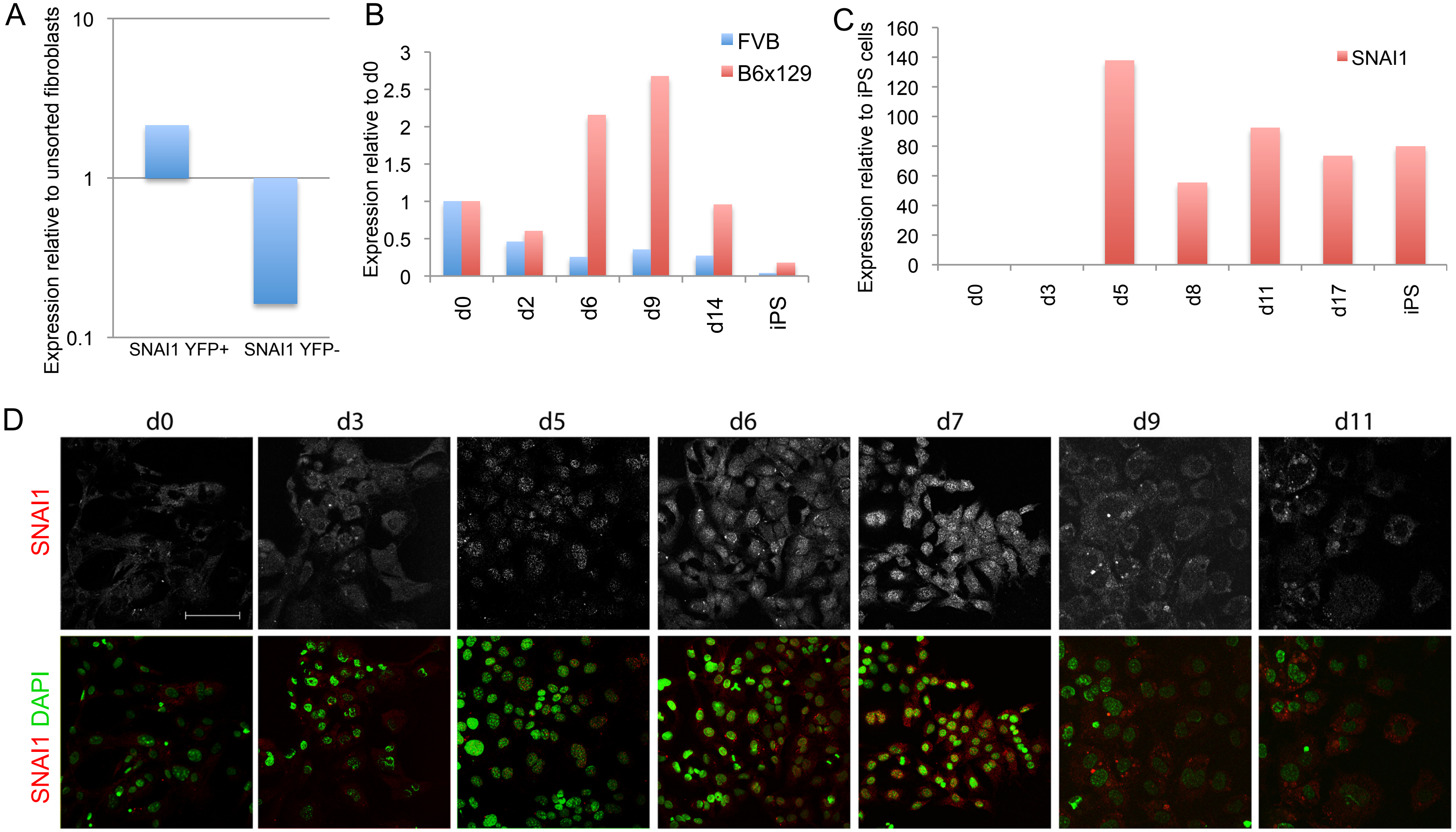

E

**Reprogramming efficiency by cell type**	**Fibroblast**	**Keratinocyte**	**Splenocytes**
From iOSKM/rtTA mice	0.3%	0.02%	0.01%

Figure S3. Endogenous *SNAI1* expression during reprogramming **(Relates to Figure 3)**.
A. Validation of knock-in reporters. Early passage MEFs from *SNAI1*-YFP knockin mice were sorted for YFP positive and negative fractions. qRT-PCR of resulting cells is shown for *SNAI1*, normalized to unsorted fibroblasts. n=2 biological replicates.
B. Time course of *SNAI1* expression during mouse reprogramming in FVB and B6x129 strains. MEFs from mouse strain B6x129 or FVB were retrovirally reprogrammed, RNA isolated at several timepoints, and qPCR for *SNAI1* performed. n=1.
C. Time course of gene expression during mouse peripheral blood reprogramming. Peripheral blood from iOSKM mice was reprogrammed by addition of Dox, and samples harvested at intervals were analyzed for *SNAI1* expression. n=1.
D. Endogenous SNAI1 expression during mouse reprogramming. iOSKM fibroblasts were reprogrammed by addition of Dox, and labeled by immunofluorescence for SNAI1 at the time points indicated.
E. Reprogramming efficiency (%) from inducible mice from three tissues. N=2-5, 1-3 biological replicates.

Figure S4. Let-7 expression during reprogramming.

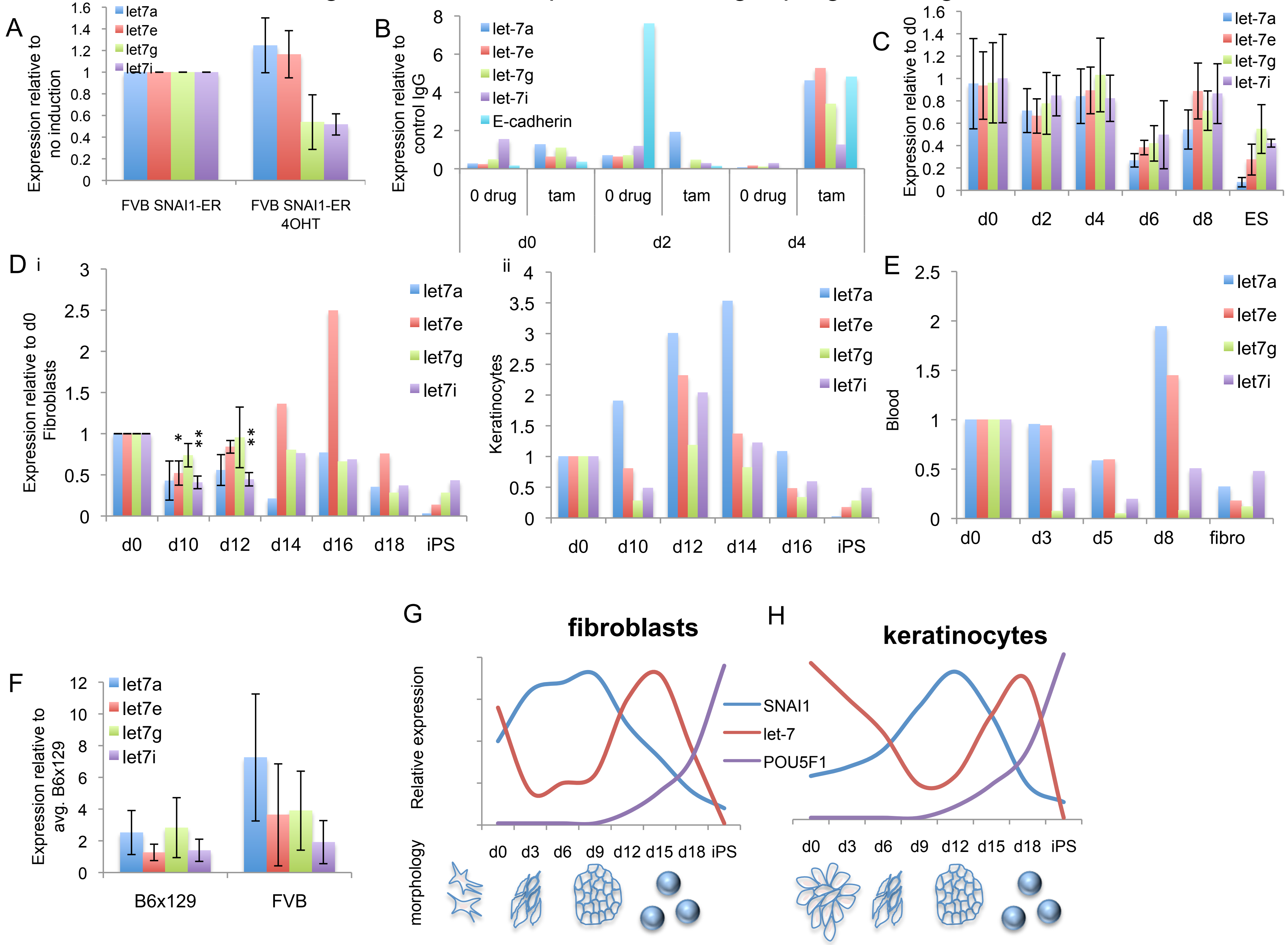

Figure S4. Let-7 expression during reprogramming or SNAI1 manipulation (Relates to Figure 4).

A. Let-7 expression upon SNAI1 overexpression after 10 days of tamoxifen treatment in FVB fibroblasts (n=4, 3 biological replicates) analyzed by TaqMan qPCR.

B. FVB Fibroblasts expressing SNAI1-ER (see figure 2)+/- tamoxifen were reprogrammed and samples collected on d0,2,and 4. ChIP was performed using anti-SNAI1 and binding to let-7 promoters was tested by qPCR. Normalization was to control IgG.

C. Let-7 expression in samples from Figure S1A fibroblasts; n=3 technical replicates.

D. Let-7 expression during the late stages of reprogramming. cDNA prepared from RNA isolated from samples on days 10, 12, 14, 16, 18 of fibroblast (i, n=3-5 biological replicates), or keratinocyte (ii, n=1) reprogramming was analyzed by TaqMan qPCR for let-7 family members; normalization is to day 0.

E. Let-7 expression in peripheral blood reprogramming. RNA samples from days 0, 3, 5, and 8 after dox addition to peripheral blood was analyzed by TaqMan qPCR for let-7 family members; n=1. Normalization is to d0 cells.

F. Let-7 expression in mouse fibroblasts from different mouse strains. RNA from untreated fibroblasts was analyzed for levels of let-7 family members by TaqMan qPCR. Normalization is to the average of the B6x129 samples. n=4 (FVB), 3 (B6x129) (3 biological replicates).

G. In fibroblasts, expression of *SNAI1* increases (blue), and let-7 decreases (red) early in reprogramming. Thereafter, pluripotency factors such as OCT4/*POU5F1* (purple) increase.

H. In keratinocytes, a similar pattern occurs, with slower kinetics.

Table 1. Primers used for qRT-PCR

Gene	Forward	Reverse	Species
Actin	TGA AGT GTG ACG TGG ACA TC	GGA GGA GCA ATG ATC TTG AT	human
GAPDH	ACC ACA GTC CAT GCC ATC AC	TCC ACC ACC CTG TTG CTG TA	mouse
POU5F1	CCT CAC TTC ACT GCA CTG TA	CAG GTT TTC TTT CCC TAG CT	human
	TCT TTC CAC CAG GCC CCC GGC TC	TGC GGG CGG ACA TGG GGA GAT CC	mouse
Snai1	CAC TAT GCC GCG CTC TTT C	GGT CGT AGG GCT GCT GGA A	human
	TCT GAA GAT GCA CAT CCG AAG CCA	AGA CTC TTG GTG CTT GTG GAG CAA	mouse
Snai2	ACT ACA GCG AAC TGG ACA CAC ACA	ACT ACA GCG AAC TGG ACA CAC ACA	mouse
Vimentin	TCT ACG AGG AGG AGA TGC GG	GGT CAA GAC GTG CCA GAG AC	mouse

Supplementary Experimental Procedures:

Mice and Cells: All mouse studies were approved by the Boston Children's Hospital IACUC, and were done in accordance with institutional and national standards and regulations. Mice used in these studies were obtained from the Weinberg lab (*SNAI1*-YFP knockin, mixed Black6/129 background), Jaenisch lab (iOSKM/rtTA, Black6/129 background (Carey et al., 2010), now available from Jackson Labs strain 011004). MEFs were isolated from e14.5 embryos (B6x129, iOSKM, *SNAI1*-YFP, iLet-7s (Zhu et al., 2011), or chimeric mice), or purchased from Chemicon (FVB and B6). iLet-7s mice overexpress Let-7g upon doxycycline treatment. Mouse keratinocytes were isolated from neonatal mice by overnight 5mg/ml Dispase (Stem Cell Technologies) digestion of skin followed by TrypLE select (invitrogen) incubation of epidermis, and were cultured in CnT-07 medium (Cell-N-Tec, ZenBio), and reprogrammed at first passage. Tail tip fibroblasts were cultured from pups or adult mice and used at low passage number. Mouse peripheral blood from iOSKM/rtTA mice (Carey et al., 2010) was obtained by retroorbital bleeding and isolation of buffy coats after settling over 1% dextran and hypotonic red blood cell lysis, and culturing in mouse ESC media with addition of mSCF (50ng/ml), hTPO (20ng/ml), mIL3, mIL6 and Flt3 ligand (10ng/ml). Chimeric mice were created by injection of NP-iPS cells harboring integrated proviruses carrying the four reprogramming factors under doxycycline control (B6x129 background) into blastocysts, which were transferred into pseudopregnant females. Fibroblasts were cultured in DMEM containing 10% FCS, 2mM L-glutamine, 1x penicillin/streptomycin (from 100x stock, Invitrogen), and 100uM 2-mercaptoethanol. Mouse ESC or iPS cell cultures

were cultured in similar media except with 15% FCS, 0.1mM nonessential amino acids (Invitrogen), and 1000U/ml ESGRO (Millipore). Human ESC or iPS cell cultures were grown with media containing DMEM/F12 with 20% knockout serum replacement, 0.1mM non-essential amino acids, 1x penicillin/streptomycin (from 100x stock, Invitrogen), 1mM L-glutamine, 50uM 2-mercaptoethanol, and 10ng/ml bFGF (Invitrogen).

Generation of reporter mouse strain: Reporter mouse strain for the expression of SNAI1 (encoding Snail), *SNAI1*-YFP, was constructed by the following procedure. A gene cassette encoding internal ribosomal entry sequence (IRES), Venus – a mutant of yellow fluorescent protein (YFP; Nagai et al. 2002, Nat Biotechnol 20, 87), and SV40 polyadenylation (polyA) sequence, together with a floxed-neor cassette, were inserted between the termination codon and the endogenous polyA sequence of each locus, doing so by the homologous recombination in v6.5 ES cells (C57BL/6 x 129S4/SvJae Hybrid). The neomycin-resistant clones of v6.5 cells were screened by PCR and Southern blotting. Following the generation of knock-in mice, floxed-neor cassette was removed from these mice by crossing them with CMV-Cre mice.

Genotyping: Mouse genotyping was done by PCR analysis of tail DNA (primers available upon request) using DNeasy kits (Qiagen), or by Transnetyx assay.

Fluorescence microscopy: Reprogrammed cells were plated on coverslips on d0 or d3 (mouse) or d5 (human) and cultured for an additional 1-5 days, then fixed in ice cold methanol followed by antibody labeling as follows: 30min incubation in permeabilization solution (PS)+ (0.05% saponin, 10mM HEPES, 10mM glycine, 10% goat serum (Invitrogen) in DMEM), 30min incubation in primary antibody diluted in PS+, 30min incubation in secondary antibody in PS+, 5min incubation in 300nM DAPI (Invitrogen), and mounting on slides with ProLong antifade reagent (Invitrogen). Cells were visualized on a Zeiss 510 confocal microscope equipped with a 40x objective.

Antibodies: Anti-SNAI1 (H-130, for immunofluorescence) was from Santa Cruz, or ab85931 from Abcam (for Western blot). Secondary antibodies were goat anti-mouse, -rat, or -rabbit Alexa Fluor 488, 568, and 647 dyes, or for Western blot, goat anti-rabbit HRP (Invitrogen).

Antibody labeling of reprogramming plates: After fixation in 4% PFA, plates were labeled with biotinylated primary antibodies and streptavidin-horseradish peroxidase (Biolegend), and visualized by diaminobenzidine reaction (Vector labs).

Reprogramming: Viral-mediated mouse reprogramming was by spinoculation of 10e5 cells per well of 6 well plate 12-24h after plating with pMX-Oct4, Sox2, Klf4, and pEYK-c-Myc viral supernatants with protamine sulfate (Sigma-Aldrich) at 6ug/ml, 2500rpm for 90min at 25°C. Individual wells of bulk cultures were harvested for assays. Colonies were counted on d21-35 (mouse) or d28-42 (human) by the following criteria: 1) colony morphology (smooth refractive well-defined borders), 2) SSEA-1 (mouse) or Tra1-60 (human, both from eBioscience) labeling. Efficiency calculations were made by counting colonies (ImageJ) of all conditions fixed on the same day according to the following formula: colonies per 100K cells = (100000 x colonies counted)/number of cells replated on d3 (mouse) or d5 (human). Non-normalized colony counts (Figure 3B) compared raw numbers of colonies obtained from an equal number of starting cells. For human reprogramming, tamoxifen alone decreased the efficiency of reprogramming (Figure S2F); efficiencies included a tamoxifen correction factor as follows: efficiency*(control efficiency without tamoxifen/control efficiency with tamoxifen).

Lentiviral knockdown: Lentivirus containing knockdown constructs was produced by co-transfection of 293T cells with lentiviral constructs, gag/pol, and VSV-G using Fugene6 (Roche). Supernatants 48 and 72 hours after transfection were harvested, filtered and stored at -80oC. Target cells were transduced by spinoculation of unconcentrated supernatants as for reprogramming. Stable lines were selected for at least 1 week. pLK0.1-puromycin (puro)-based constructs against SNAI1 were obtained from Sigma (SHDNA-NM_011427, SHDNA-NM_011415). Lentivirus containing KD constructs was

produced as described (Onder et al., 2012) and transduced by spinoculation as for reprogramming. Stable cell lines were selected by addition of Puro at 1ug/ml on day 2 following transduction, continuing for at least 1 week.

Retroviral overexpression: pWZL-based vectors expressing Twist- or SNAI1-ER, or a control empty vector (Mani et al., 2008) were used to generate virus, and transduced by spinoculation as above. Cells were induced for at least 10 days by addition of 4-OH-tamoxifen (Sigma-Aldrich) at 20nM, followed by reprogramming. Lines were selected starting on day 2 after transduction using 5 ug/ml Blasticidin and continuing for at least one week.

Quantitative real-time PCR (qRT-PCR): RNA was isolated using Trizol or RNeasy mini- or micro kits (Qiagen), and cDNA was generated from 1ug RNA using the Superscript III first strand synthesis kit (Invitrogen). Results were normalized by comparison to GAPDH (mouse) or Actin B (human). qRT-PCR was performed using SYBR green master mix (Stratagene) on a Stratagene Mx3000P. Primer sequences are listed in Supplementary Table 1 online. microRNA was isolated using Trizol (Invitrogen) or miRNeasy (Qiagen), cDNA was generated from 100ng RNA using TaqMan microRNA reverse transcription kit (Applied Biosystems), and levels of mature miRNA were detected using commercially available TaqMan

probes (Applied Biosystems) per manufacturer's instructions with sno142 RNA as internal standards for normalization. miR qPCR was done as described (Viswanathan et al., 2008).

Chromatin immunoprecipitation assays: ChIP analyses were carried out on chromatin extracts according to manufacturer's specifications (MAGnify ChIP – Invitrogen) with anti-SNAI1 from Abcam, ab85931 and from Cell Signaling Technology, L70G2. Data are represented as fold enrichment with respect to control antibody (IgG), normalized to background signal of specific antibody over a negative genomic region. Let-7a-2, let-7g, let-7e, and let-i were analyzed, with oligos designed from a region comprising 2kb upstream of the stem-loop of the first member of the cluster. Primer pairs were tested for amplification efficiency by generating a standard curve over five dilutions of input sample. Data are representative of three independent experiments. Primer sequences are available upon request.

Statistics: Statistical analysis was performed using the Student's two-tailed t-test. *, $p<0.05$; **, $p<0.01$; ***, $p<0.001$. Error bars indicate SEM.

Supplemental references.

Carey, B.W., Markoulaki, S., Beard, C., Hanna, J., and Jaenisch, R. (2010). Single-gene transgenic mouse strains for reprogramming adult somatic cells. Nat Methods *7*, 56-59.

Mani, S.A., Guo, W., Liao, M.J., Eaton, E.N., Ayyanan, A., Zhou, A.Y., Brooks, M., Reinhard, F., Zhang, C.C., Shipitsin, M., *et al.* (2008). The epithelial-mesenchymal transition generates cells with properties of stem cells. Cell *133*, 704-715.

Onder, T.T., Kara, N., Cherry, A., Sinha, A.U., Zhu, N., Bernt, K.M., Cahan, P., Marcarci, B.O., Unternaehrer, J., Gupta, P.B., *et al.* (2012). Chromatin-modifying enzymes as modulators of reprogramming. Nature *483*, 598-602.

Viswanathan, S.R., Daley, G.Q., and Gregory, R.I. (2008). Selective blockade of microRNA processing by Lin28. Science *320*, 97-100.

Zhou, B.P., Deng, J., Xia, W., Xu, J., Li, Y.M., Gunduz, M., and Hung, M.C. (2004). Dual regulation of Snail by GSK-3beta-mediated phosphorylation in control of epithelial-mesenchymal transition. Nat Cell Biol *6*, 931-940.

Zhu, H., Shyh-Chang, N., Segre, A.V., Shinoda, G., Shah, S.P., Einhorn, W.S., Takeuchi, A., Engreitz, J.M., Hagan, J.P., Kharas, M.G., *et al.* (2011). The Lin28/let-7 axis regulates glucose metabolism. Cell *147*, 81-94.
