# Supplementary material for: The Epithelial-Mesenchymal Transition Factor SNAIL Paradoxically Enhances Reprogramming
Source: Stem Cell Reports. 2014 Oct 11;3(5):691–8. doi: 10.1016/j.stemcr.2014.09.008 (PMC4235745; doi:10.1016/j.stemcr.2014.09.008)
Supplement: Document S2. Article plus Supplemental Information [file mmc2.pdf]

Stem Cell Reports
Report
ISSCR
OPEN ACCESS

The Epithelial-Mesenchymal Transition Factor SNAIL Paradoxically Enhances Reprogramming

Juli J. Unternaehrer,[1,2,3,7,*] Rui Zhao,[1,2,3,8] Kitai Kim,[1,2,3,9] Marcella Cesana,[1,2,3] John T. Powers,[1,2,3] Sutheera Ratanasirintrawoot,[1,2,3] Tamer Onder,[1,2,3,10] Tsukasa Shibue,[4,5] Robert A. Weinberg,[4,5,6] and George Q. Daley[1,2,3]
[1]Division of Pediatric Hematology/Oncology, Stem Cell Transplantation Program, Manton Center for Orphan Disease Research, Howard Hughes Medical Institute, Children's Hospital Boston and Dana Farber Cancer Institute
[2]Department of Biological Chemistry and Molecular Pharmacology, Harvard Medical School
[3]Harvard Stem Cell Institute
Harvard University, Cambridge, MA 02138, USA
[4]Whitehead Institute for Biomedical Research, 9 Cambridge Center, Cambridge, MA 02142, USA
[5]Ludwig Center for Molecular Oncology
[6]Department of Biology
Massachusetts Institute of Technology, 77 Massachusetts Avenue, Cambridge, MA 02139, USA
[7]Present address: Division of Biochemistry, Loma Linda University, 11085 Campus Street, Loma Linda, CA 92354, USA
[8]Present address: Department of Biochemistry and Molecular Genetics and UAB Stem Cell Institute, Birmingham, AL 35294, USA
[9]Present address: Cancer Biology & Genetics Program, Center for Cell Engineering, Center for Stem Cell Biology, Department of Cell and Developmental Biology, Weill Medical College of Cornell University, Sloan-Kettering Institute, New York, NY 10065, USA
[10]Present address: Koc University School of Medicine, 34450 Istanbul, Turkey
*Correspondence: junternaehrer@llu.edu
http://dx.doi.org/10.1016/j.stemcr.2014.09.008
This is an open access article under the CC BY license (http://creativecommons.org/licenses/by/3.0/).

SUMMARY

Reprogramming of fibroblasts to induced pluripotent stem cells (iPSCs) entails a mesenchymal to epithelial transition (MET). While attempting to dissect the mechanism of MET during reprogramming, we observed that knockdown (KD) of the epithelial-to-mesenchymal transition (EMT) factor *SNAI1 (SNAIL)* paradoxically reduced, while overexpression enhanced, reprogramming efficiency in human cells and in mouse cells, depending on strain. We observed nuclear localization of SNAI1 at an early stage of fibroblast reprogramming and using mouse fibroblasts expressing a knockin *SNAI1*-YFP reporter found cells expressing SNAI1 reprogrammed at higher efficiency. We further demonstrated that SNAI1 binds the let-7 promoter, which may play a role in reduced expression of let-7 microRNAs, enforced expression of which, early in the reprogramming process, compromises efficiency. Our data reveal an unexpected role for the EMT factor SNAI1 in reprogramming somatic cells to pluripotency.

INTRODUCTION

Reprogramming somatic cells to induced pluripotent stem cells (iPSCs) holds great promise for disease modeling and therapeutic applications. Among the challenges that remain is the extended time frame and variable efficiency of transcription factor-based reprogramming; in most cases, fewer than 0.05% of the target population will form bona fide iPSC colonies (Chan et al., 2009; Takahashi et al., 2007). Improving our knowledge of the mechanisms of reprogramming should facilitate more efficient reprogramming.

Studies aimed at elucidating the mechanism of reprogramming somatic cells to pluripotency have revealed a multistep process in which exogenous OCT4, SOX2, KLF4, and MYC (OSKM) expression initiates events resulting in endogenous expression of pluripotency factors and a stable iPSC phenotype. Fibroblasts, the somatic cells most often targeted for reprogramming, are quintessential mesenchymal cells, whereas embryonic stem (ES) or iPSCs express epithelial markers. Completion of the prolonged reprogramming process entails upregulation of epithelial factors and downregulation of mesenchymal factors—a classic mesenchymal to epithelial transition (MET) (Samavarchi-Tehrani et al., 2010). KLF4 has been shown to induce E-cadherin and other epithelial factors, whereas SOX2 and OCT4 downregulate SNAI1 (Li et al., 2010). Thus, a mechanistic link between MET and fibroblast reprogramming has been proposed.

However, considerable data suggest that reprogramming is not a simple MET from start to finish. A sequential application of reprogramming factors, shown to induce an early epithelial-to-mesenchymal transition (EMT), improves efficiency, as does early transforming growth factor-β (TGF-β) treatment or Slug expression (Liu et al., 2013). Wnt signaling, which promotes EMT, enhances reprogramming if activated early in the process (Marson et al., 2008). Inhibitors of TGF-β signaling, which promote MET, can enhance reprogramming if administered concomitant with OSKM expression (Ichida et al., 2009) but antagonize reprogramming if administered in the 3 preceding days (Maherali and Hochedlinger, 2009). Taken together, these data imply

that induction of mesenchymal fates may play a positive role in the reprogramming process. The role of EMT factors in induction of pluripotency has not been extensively explored.

The transcription factor SNAI1 represses epithelial factors such as E-cadherin, and its expression is sufficient for EMT (Cano et al., 2000). It exerts a positive influence on the expression of stemness factors, including SOX2 and KLF4, via effects on microRNAs (miRs) (Garibaldi et al., 2012), and has been shown to decrease proliferation while preventing apoptosis (Vega et al., 2004).

LIN28, a regulator of miR biogenesis and an alternative reprogramming factor (Viswanathan et al., 2008; Yu et al., 2007), inhibits the processing and maturation of let-7 and is in turn a let-7 target (Rybak et al., 2008). Mature let-7 family miRs, regulators of developmental timing (Ambros, 2011), are absent in pluripotent cells and are expressed at high levels in differentiated cell populations (Viswanathan et al., 2008). Let-7 inhibits expression of pluripotency factors (including LIN28, c-MYC, and SALL4) (Melton et al., 2010) and cell cycle regulators critical for the ES cell phenotype (such as CDK6, CDC25A, and cyclin D) (reviewed in (Mallanna and Rizzino, 2010). Expression of let-7 miRs can promote differentiation of pluripotent stem cells, and a let-7 inhibitor promotes dedifferentiation; thus, let-7 downregulation is likely essential to reprogramming (Melton et al., 2010). Let-7 inhibition stimulates OSK reprogramming efficiency (without c-MYC) to the same extent as does c-MYC, and forced let-7 expression decreases reprogramming efficiency (Worringer et al., 2014). Moreover, a connection between EMT factors and the transcriptional regulation of let-7 has been reported (Chang et al., 2011; Kong et al., 2010; Li et al., 2009).

Here we set out to evaluate the roles of transcriptional regulators associated with EMT in iPSC reprogramming. We were intrigued by data from published reports that EMT factors (including *SNAI1*) increased during the early stages of reprogramming (Mikkelsen et al., 2008; Samavarchi-Tehrani et al., 2010). We were likewise perplexed by the continued expression of this and other EMT factors at time points when genes downregulated by them were strongly expressed. Thus, we examined the roles of SNAI1 during reprogramming.

RESULTS

Effects of SNAI1 on Reprogramming

We investigated expression levels of *SNAI1* and pluripotency markers *POU5F1* (OCT4) and *LIN28A* during OSKM reprogramming (Figures S1A and S1B available online) of fibroblasts and keratinocytes and found that *SNAI1* expression was variably increased through intermediate stages in reprogramming in both mouse and human cultures. Our expression data were consistent with published microarrays showing, in fibroblasts, upregulation of *SNAI1* and other EMT factors early in mouse reprogramming, while reduced *SNAI1* levels were not observed until day 8 (Mikkelsen et al., 2008; Samavarchi-Tehrani et al., 2010). Although the kinetics of reprogramming are variable between experiments, we consistently observed early upregulation of *SNAI1*, leading us to hypothesize that it might play a positive role.

To explore the role of *SNAI1*, we knocked it down in murine fibroblasts and evaluated the effects on the efficiency of reprogramming. We confirmed that *SNAI1* was knocked down by quantitative PCR (qPCR) and immunoblot (Figures S1C and S1D). Unexpectedly, we observed that knockdown (KD) caused a trend toward decreased rather than increased reprogramming efficiency in two different mouse strains, C57BL/6 × 129 (B6×129) and mouse strain Friend Virus B (FVB) (Figures 1A and 1B). To enable analysis of live colonies, we used colony morphology and stage-specific embryonic antigen 1 (SSEA-1) expression as our indicator of successful reprogramming, after showing that numbers based on Nanog expression in fixed colonies correlated with those obtained by SSEA-1 labeling (Figure S1E). To investigate whether EMT factors play similar roles in reprogramming of human somatic cells, we employed a "secondary" reprogramming system in which fibroblasts were differentiated from iPSCs carrying doxycycline (dox)-inducible reprogramming factors (Figure S2A) (Kim et al., 2011). In secondary human fibroblasts (D2F), KD of *SNAI1* likewise compromised reprogramming (Figures 1A, 1B, and S1C).

We then enforced expression of SNAI1 by tamoxifen (TMX) induction of an estrogen receptor (ER) fusion construct prior to the initiation of reprogramming (Mani et al., 2008). In fibroblasts, levels of expression of EMT factors are relatively low, and they increase upon TGF-β treatment (Figure S2B). While constitutive SNAI1 overexpression during reprogramming has been shown to decrease efficiency (Li et al., 2010), its effects during the early timeframe have not been tested. We monitored rates of proliferation since SNAI1 is known to play a role in cell cycle regulation (Vega et al., 2004). Upon TMX addition to control versus SNAI1-expressing cells, changes in proliferation rates were not significant (Figure S2C). SNAI1 expression in the nucleus increased upon TMX treatment (Figures 2A and S2D). Cells in which SNAI1 had been induced were then reprogrammed by viral transduction following cessation of TMX. In MEFs from FVB mice, overexpression of SNAI1 prior to reprogramming increased efficiency, while in B6×129, a strain with high baseline reprogramming efficiency, SNAI1 did not further augment

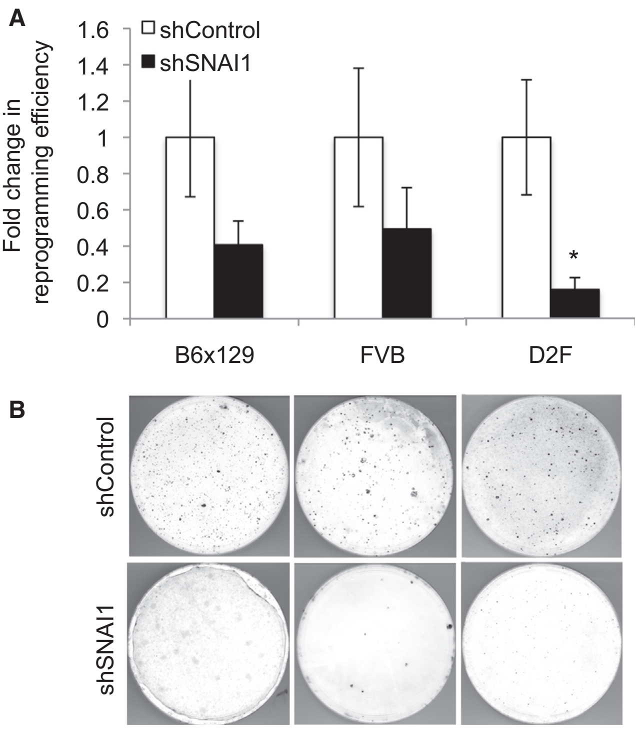

Figure 1. KD of *SNAI1* in Fibroblasts Decreases Reprogramming Efficiency

(A) In MEFs or D2F, *SNAI1* was knocked down by three different shRNAs individually, followed by retroviral reprogramming or dox addition, and colonies were counted based on morphology and SSEA-1 or Tra1-60 labeling after 21 or 28 days, respectively, normalized to cell number on day 3. Shown is fold change relative to control (scrambled shRNA); bars indicate shControl (white) and shSNAI1 (black). n = 10–22 in four to nine biological replicates. Error bars show SEM. *$p < 0.05$. B6×129: $p = 0.06$, FVB: $p = 0.08$. See also Figure S1.

(B) SSEA-1 labeling of B6×129 (left), FVB (middle), or Tra1-60 labeling of D2F (right); control shRNA (upper) and *SNAI1* KD (lower) are shown.

reprogramming (Figures 2B and S2H). *SNAI1* expression in the two strains was similar (Figure S2I).

We reasoned that if SNAI1 plays a role in reprogramming we should observe a more dramatic effect on the reprogramming of epithelial cells, such as keratinocytes, because their intrinsic level of expression of *SNAI1*, although not absent, is lower than in fibroblasts (Figure S2F). To test this and to discern the effect in human cells, we overexpressed SNAI1 via ER fusion in human fibroblasts and keratinocytes prior to reprogramming and confirmed TMX-induced nuclear translocation (Figure S2E). Overexpression of SNAI1 in both cell types caused an enhancement of colony formation, with a more pronounced effect in epithelial cells (Figure 2C), confirming that both mesenchymal and epithelial cell types reprogram more efficiently when SNAI1 is expressed. Accordingly, with respect to fibroblasts, SNAI1 expression is more markedly upregulated in keratinocytes, presumably due to lower starting levels (Figures S1A and S1B).

Cells Expressing Endogenous SNAI1 Reprogram More Efficiently

Before reprogramming, MEFs expressed low levels of *SNAI1* (Figure 3A, upper panel). Early in the reprogramming process (days 1–7), endogenous SNAI1 became localized to the nucleus (Figure 3A, lower panel; day 5 shown; Figure S3D).

To test whether cells expressing endogenous SNAI1 are more efficiently reprogrammed, we isolated cells from mice with a knockin reporter construct that enables selection for cells expressing SNAI1 by virtue of yellow fluorescent protein coexpression from an internal ribosomal entry site (T.S. and R.A.W., unpublished data). We noted varying proportions of YFP-positive cells in tail tip fibroblasts and mouse embryonic fibroblasts (MEFs). Sorted *SNAI1*-YFP-positive and -negative populations expressed higher and lower levels of *SNAI1* mRNA, respectively (Figure S3A). YFP-positive fractions showed an increased reprogramming efficiency for SNAI1 (6.5×) as compared with negative populations (Figure 3B). We found that upon culture fewer YFP-negative than YFP-positive cells resulted, either because of proliferative or cell death differences, but after normalizing for cell number differences (as described in Experimental Procedures), enhancement was still seen for SNAI1 (2.6×, Figure 3C).

We observed an increase in *SNAI1* expression early in reprogramming in B6×129, but not in FVB (Figure S3B), the strain in which SNAI1 overexpression increased efficiency. *SNAI1* expression was also seen in mouse peripheral blood reprogramming (Figure S3C). Thus, an increase in *SNAI1* expression was seen across mesenchymal, epithelial, and peripheral blood cell types and could be observed by monitoring RNA or protein.

SNAI1 Expression Is Temporally Associated with Let-7 Downregulation

Next, we explored the potential mechanism by which SNAI1 enhances reprogramming, noting the references that link EMT with downregulation of the let-7 family of tumor suppressor miRs (Chang et al., 2011; Kong et al., 2010; Li et al., 2009; Yang et al., 2012). Using inducible Snail ER, we observed downregulation of let-7 after 7 days of TMX treatment in mouse fibroblasts (Figures 4A and S4A). KD of *SNAI1* resulted in increased let-7 expression (Figure 4B). Chromatin immunoprecipitation (ChIP) confirmed that SNAI1 binds the promoters of several let-7 family members during early stages of reprogramming in B6×129 fibroblasts (Figure 4C) and in FVB overexpressing

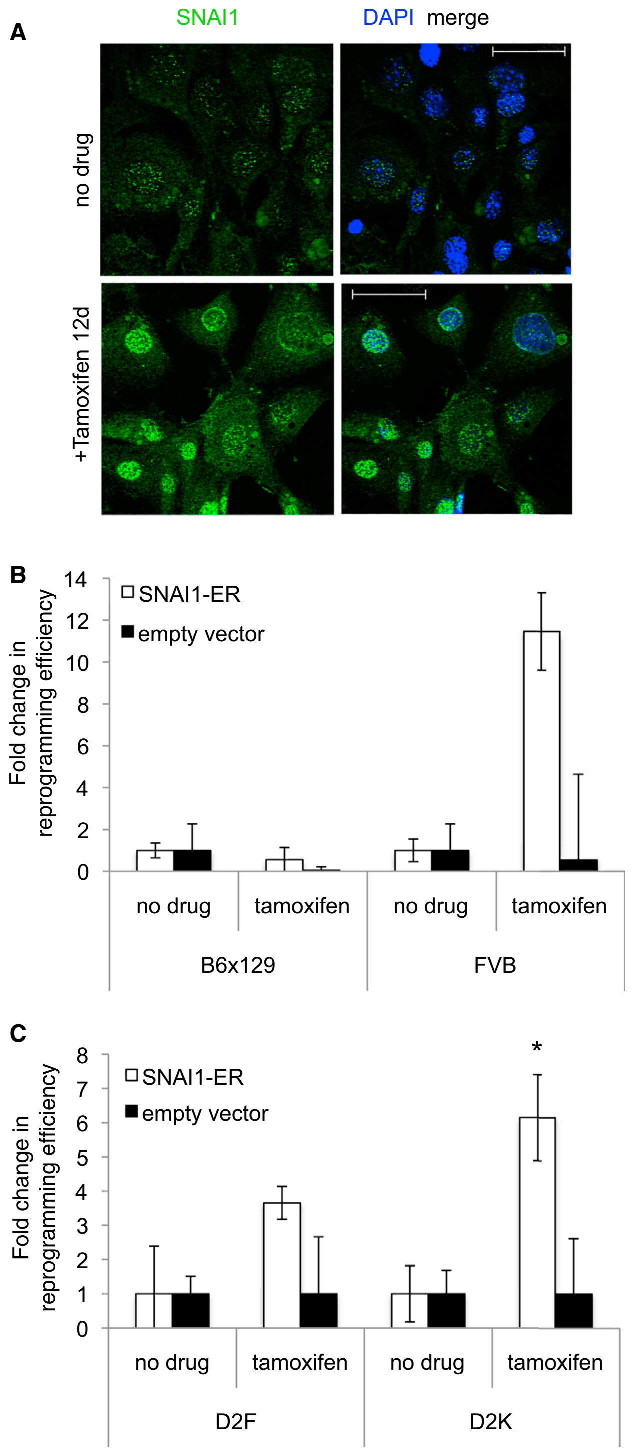

Figure 2. Overexpression of SNAI1 via ER Fusion Improves Reprogramming Efficiency

(A) A B6×129 line expressing SNAI1-ER was created by retroviral transduction. Cells were treated with TMX for 12 days and fixed and labeled. With (lower) or without induction (upper); SNAI1 labeling, green; DAPI, blue; 40×, scale bar represents 50 μm.

(B) Mouse fibroblasts of indicated strain were reprogrammed and efficiency calculated based on colony morphology and SSEA-1 labeling. (C) D2F or D2K were reprogrammed as in B and Tra 1-60+ colonies labeled. (B and C) White bars, SNAI1-ER; black, empty vector; fold increase over no TMX is shown. n = 3–14, 2–6 biological replicates. Error bars in (B) and (C) show SEM. *$p < 0.05$.
See also Figure S2.

SNAI1-ER more so than without induction (Figure S4B). Upon TMX treatment of SNAI1-ER expressing fibroblasts (without reprogramming), SNAI1 binding to let-7 members similarly increases (Figure 4D).

We evaluated expression of let-7 during OSKM-induced reprogramming and found let-7a, let-7e, let-7g, and let-7i decreased in both fibroblasts and keratinocytes in the early phase (Figures 4E, 4F, S4C (parallel fibroblast data for Figure S1A), and S4D). A similar trend can be seen in the case of mouse peripheral blood (Figure S4E). To understand the role of let-7 in reprogramming, we expressed let-7 in MEFs from a strain of mice carrying a dox-inducible transgene at various stages of reprogramming (Zhu et al., 2011). We found that let-7 overexpression compromised efficiency when done during the first, but not the second, 7 days of reprogramming (Figure 4G). We also noted a trend toward higher expression of several let-7 members in FVB than in B6×129 strain prior to reprogramming, correlating high expression with augmentation of reprogramming efficiency upon SNAI1 overexpression (Figure S4F). These data suggest downregulation of the let-7 miRs as a possible mechanism by which SNAI1 influences reprogramming (diagrammed in Figures 4H, S4G, and S4H).

DISCUSSION

Fibroblasts are the typical starting population for somatic cell reprogramming, and prior studies have indicated that reprogramming involves an MET. Paradoxically, however, transcription factors associated with EMT are expressed early in the reprogramming process and are not downregulated until the later stages (Samavarchi-Tehrani et al., 2010). While previously it was reported that keratinocytes could be reprogrammed with higher efficiency because of their pre-existing epithelial status (Maherali et al., 2008), a side-by-side comparison between cell types has not been done. In a secondary reprogramming system that enables direct comparison, we found mouse keratinocytes were reprogrammed less efficiently (0.02%) than fibroblasts (0.3%) (Figure S3E), and we have discovered ectopic expression of the EMT factor SNAI1 during early stages of reprogramming enhances efficiency in keratinocytes, an epithelial cell type. Thus, the effect of expression of EMT factors in the initial phase of reprogramming is not limited

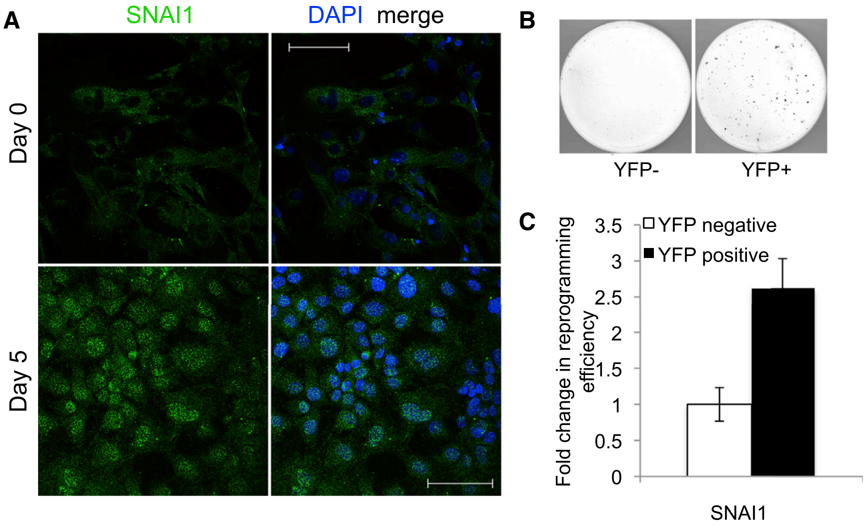

Figure 3. SNAI1 Expression, Upregulated Early in Reprogramming, Predicts Higher Reprogramming Efficiency

(A) On days 0 and 5 of reprogramming, B6× 129 fibroblasts were labeled with anti-SNAI1 (green, left panels) and DAPI (blue, overlaid with green in right panels); 40×, scale bar represents 50 μm.

(B) YFP-positive and -negative sorted MEFs from *SNAI1*-YFP knockin mice were retrovirally reprogrammed. SSEA1+ colonies were counted on day 21; representative images are shown.

(C) Quantification of colonies, normalized for cell number on day 3. n = 7–19, 5 biological replicates. Error bars show SEM.

See also Figure S3.

to mesenchymal target cell populations, but also occurs in epithelial cells, suggesting mesenchymal factor expression is an important aspect of reprogramming independent of starting cell type. Manipulating SNAI1 has led us to a multistep model of reprogramming whereby mesenchymal factors are expressed early and contribute to the reprogramming-amenable state (Koche et al., 2011), and only thereafter are pluripotency factors expressed en route to the pluripotent state seen in iPSCs (Figure 4H). Our data are in agreement with a recent study (Liu et al., 2013), which reported early enhancement of the mesenchymal state increased reprogramming efficiency.

A role for let-7 in reprogramming has been established since its inhibition increases reprogramming efficiency (Melton et al., 2010). As shown here, SNAI1 binds several let-7 promoters, and *SNAI1* expression is associated temporally with downregulation of let-7 miRs early in reprogramming, consistent with prior evidence that EMT factors suppress let-7 expression in cancer (Yang et al., 2012). Moreover, overexpressing SNAI1 in a poorly reprogramming strain augments both reprogramming efficiency and SNAI1 binding to the let-7 promoter, suggesting SNAI1 regulation of let-7 may be the basis for enhanced reprogramming efficiency. The downregulation of let-7 transcription by SNAI1 may be associated with upregulation of LIN28 by pluripotency factors, thereby potently reversing the differentiated state. While let-7 is downregulated in the first week of reprogramming, its expression appears to recover thereafter before again diminishing to near zero in the iPSC state (Figures 4E, 4F, and S4D). We have not studied this biphasic expression pattern, but we hypothesize the second wave is extinguished by LIN28.

Prior studies have demonstrated that Twist promotes a stem cell phenotype in cancer, including self-renewal (Mani et al., 2008). We hypothesize that expression of SNAI1 might similarly promote a stem cell-like phenotype in fibroblasts and keratinocytes, moving them one step closer to dedifferentiation and making them more amenable to reprogramming. We propose that suppression of let-7 miRs is a mechanism whereby SNAI1 might be acting to confer these stem cell properties.

Building on the model proposed by Samavarchi-Tehrani et al. and Li et al. demonstrating the role of MET in reprogramming, we show during the early phases of reprogramming, mesenchymal factors are expressed, and further ectopic expression of EMT factors enhances reprogramming efficiency. Our results provide a more nuanced view of the role of EMT factors in the reprogramming of both mesenchymal and epithelial cell types. Our data are corroborated by an independent study that identified *SNAI1* in an unbiased shRNA screen as a factor that enhances conversion of pre-iPS cells to a fully reprogrammed state, thereby reinforcing the conclusion that SNAI1 acts to enhance reprogramming (Gingold et al., 2014). This improved understanding of the mechanism of reprogramming will provide strategies to improve its utility for modeling and treating disease and advance our insight into the regulation of gene expression and pluripotency.

EXPERIMENTAL PROCEDURES

Mice and Cells

All mouse studies were approved by the Boston Children's Hospital IACUC and were done in accordance with institutional and national standards and regulations. Mouse keratinocytes were isolated from neonatal mice, cultured in CnT-07 medium (Cell-N-Tec, ZenBio) and reprogrammed at first passage. Second-generation inducible iPSCs were generated from H1 human embryonic stem cell by differentiation to fibroblasts (Park et al., 2008), transduction with inducible OSKM lentiviruses (Stadtfeld et al., 2008), and dox

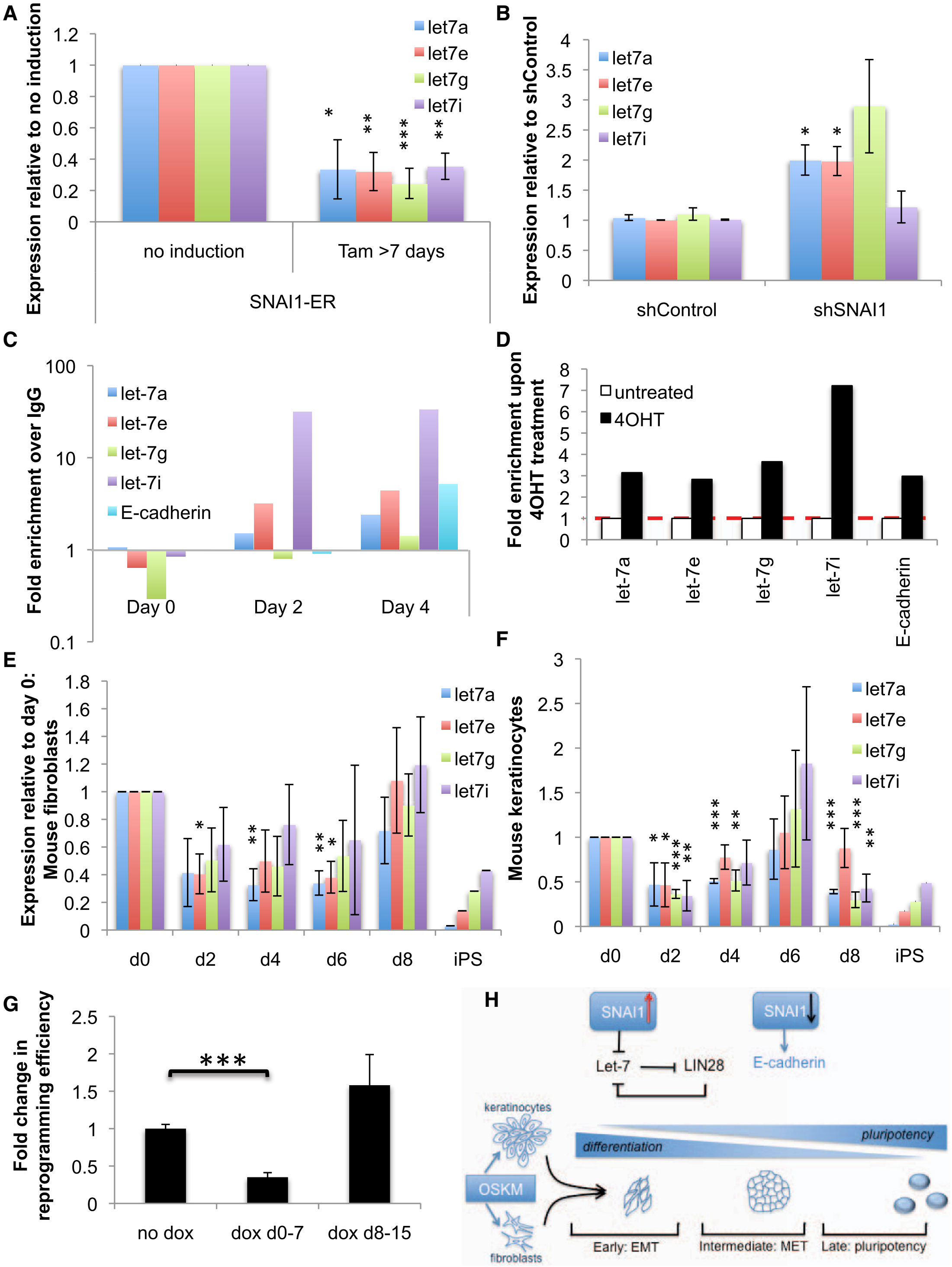

Figure 4. Snail Expression Is Temporally Associated with Downregulation of Let-7

(A) qPCR analyses on let-7 expression upon SNAI1 overexpression. After 7 or 10 days of TMX treatment, B6×129 fibroblasts stably expressing SNAI1-ER were tested for levels of expression of let-7 revealed by TaqMan qPCR (n = 3–4 biological replicates). Error bars show SEM.

(B) qPCR analyses of let-7 expression upon *SNAI1* KD. B6×129 fibroblasts with lentiviral *SNAI1* KD were analyzed for expression of let-7 (n = 3 biological replicates). Error bars show SEM.

(legend continued on next page)

induction. Keratinocytes were differentiated from iPSCs by a 6 day culture in basic fibroblast growth factor-free media followed by dissociation and 3–4 day culture in keratinocyte serum-free medium (Invitrogen) +/− retronectin (Takara Shuzo) (Kim et al., 2011).

Reprogramming

Retroviral-mediated mouse reprogramming was via pmX constructs. Second-generation inducible fibroblasts or keratinocytes were induced by addition of dox at 2 μg/ml 12–24 h after plating. In both cases, cells were replated onto irradiated MEFs (GlobalStem) 3 days (mouse) or 5 days (human) after expression of the four factors, with daily changes of mouse or human ES cell media thereafter.

Flow Cytometry

Tail tip fibroblasts or MEFs positive or negative for YFP were sorted on a FACSAria (BD). Cells were used immediately for reprogramming or qPCR.

ChIP Assays

ChIP analyses were carried out as described (Cesana et al., 2011).

SUPPLEMENTAL INFORMATION

Supplemental Information includes Supplemental Experimental Procedures, four figures, and one table and can be found with this article online at http://dx.doi.org/10.1016/j.stemcr.2014.09.008.

ACKNOWLEDGMENTS

We are grateful to Patrick Cahan, Francesca Casano, and M. Willy Lensch for insightful discussion of the manuscript; Ronald Mathieu for FACS sorting; Yuko Fujiwara and Minh Nguyen for blastocyst injection; and Kitwa Ng and Kerrianne Cunniff Schlosser for expert technical assistance. G.Q.D. is a member of the scientific advisory board of iPierian and Verastem. The work was supported by private funds of the Boston Children's Hospital Stem Cell Program and by NIH R01-GM107536. J.J.U. was supported by NIH-T32-HL07623-23.

Received: May 27, 2013
Revised: September 9, 2014
Accepted: September 9, 2014
Published: October 9, 2014

REFERENCES

Ambros, V. (2011). MicroRNAs and developmental timing. Curr. Opin. Genet. Dev. *21*, 511–517.

Cano, A., Pérez-Moreno, M.A., Rodrigo, I., Locascio, A., Blanco, M.J., del Barrio, M.G., Portillo, F., and Nieto, M.A. (2000). The transcription factor snail controls epithelial-mesenchymal transitions by repressing E-cadherin expression. Nat. Cell Biol. *2*, 76–83.

Cesana, M., Cacchiarelli, D., Legnini, I., Santini, T., Sthandier, O., Chinappi, M., Tramontano, A., and Bozzoni, I. (2011). A long noncoding RNA controls muscle differentiation by functioning as a competing endogenous RNA. Cell *147*, 358–369.

Chan, E.M., Ratanasirintrawoot, S., Park, I.H., Manos, P.D., Loh, Y.H., Huo, H., Miller, J.D., Hartung, O., Rho, J., Ince, T.A., et al. (2009). Live cell imaging distinguishes bona fide human iPS cells from partially reprogrammed cells. Nat. Biotechnol. *27*, 1033–1037.

Chang, C.J., Hsu, C.C., Chang, C.H., Tsai, L.L., Chang, Y.C., Lu, S.W., Yu, C.H., Huang, H.S., Wang, J.J., Tsai, C.H., et al. (2011). Let-7d functions as novel regulator of epithelial-mesenchymal transition and chemoresistant property in oral cancer. Oncol. Rep. *26*, 1003–1010.

Garibaldi, F., Cicchini, C., Conigliaro, A., Santangelo, L., Cozzolino, A.M., Grassi, G., Marchetti, A., Tripodi, M., and Amicone, L. (2012). An epistatic mini-circuitry between the transcription factors Snail and HNF4α controls liver stem cell and hepatocyte features exhorting opposite regulation on stemness-inhibiting microRNAs. Cell Death Differ. *19*, 937–946.

Gingold, J., Fidalgo, M., Guallar, D., Lau, Z., Sun, Z., Zhou, H., Faiola, F., Huang, X., Lee, D.-F., Waghray, A., et al. (2014). A genome-wide RNAi screen identifies opposing functions of Snai1 and Snai2 on the Nanog dependency in reprogramming. Mol. Cell. Published online September 15, 2014. http://dx.doi.org/10.1016/j.molcell.2014.08.014.

(C) ChIP analysis of endogenous SNAI1 on promoters of let-7 genes. Samples from iOSKM MEF reprogramming were harvested at days 0, 2, and 4, prepared for ChIP, and analyzed for SNAI1 binding to E boxes in the promoters of let-7a, let-7e, let-7g, and let-7i. Binding to the E-cadherin promoter functioned as a positive control. IgG, control antibody. n = 3 biological replicates; representative experiment shown.
(D) ChIP analysis of SNAI1-ER upon TMX treatment. iOSKM MEFs stably expressing SNAI1-ER (see Figure 2) were harvested before or after 10 days of induction with TMX. Binding to the promoters of let-7a, let-7e, let-7g, and let-7i was analyzed by ChIP. White bars, untreated; black bars, + TMX.
(E and F) qPCR analyses of let-7 expression during the reprogramming of iOSKM (B6×129) fibroblasts and keratinocytes. RNA samples isolated from days 0, 2, 4, 6, and 8 of fibroblast (E, n = 4–5) or keratinocyte (F, n = 3 biological replicates) reprogramming was analyzed by TaqMan qPCR for let-7. *$p < 0.05$; **$p < 0.01$; ***$p < 0.001$. Error bars show SEM. See also Figure S4.
(G) Forced expression of let-7 in reprogramming. iLet-7 MEFs were retrovirally reprogrammed with or without dox addition at days 0–7 and day 8–15. n = 3–5, 3 biological replicates. Error bars show SEM. ***$p < 0.001$.
(H) Schematic representation of hypothesis. After expression of four pluripotency factors, *SNAI1* upregulation leads to let-7 downregulation. Targeting of LIN28 and other pluripotency factors is thus removed, and the differentiated state is destabilized, allowing expression of pluripotency factors.

Ichida, J.K., Blanchard, J., Lam, K., Son, E.Y., Chung, J.E., Egli, D., Loh, K.M., Carter, A.C., Di Giorgio, F.P., Koszka, K., et al. (2009). A small-molecule inhibitor of tgf-Beta signaling replaces sox2 in reprogramming by inducing nanog. Cell Stem Cell *5*, 491–503.

Kim, K., Zhao, R., Doi, A., Ng, K., Unternaehrer, J., Cahan, P., Huo, H., Loh, Y.H., Aryee, M.J., Lensch, M.W., et al. (2011). Donor cell type can influence the epigenome and differentiation potential of human induced pluripotent stem cells. Nat. Biotechnol. *29*, 1117–1119.

Koche, R.P., Smith, Z.D., Adli, M., Gu, H., Ku, M., Gnirke, A., Bernstein, B.E., and Meissner, A. (2011). Reprogramming factor expression initiates widespread targeted chromatin remodeling. Cell Stem Cell *8*, 96–105.

Kong, D., Banerjee, S., Ahmad, A., Li, Y., Wang, Z., Sethi, S., and Sarkar, F.H. (2010). Epithelial to mesenchymal transition is mechanistically linked with stem cell signatures in prostate cancer cells. PLoS ONE *5*, e12445.

Li, Y., VandenBoom, T.G., 2nd, Kong, D., Wang, Z., Ali, S., Philip, P.A., and Sarkar, F.H. (2009). Up-regulation of miR-200 and let-7 by natural agents leads to the reversal of epithelial-to-mesenchymal transition in gemcitabine-resistant pancreatic cancer cells. Cancer Res. *69*, 6704–6712.

Li, R., Liang, J., Ni, S., Zhou, T., Qing, X., Li, H., He, W., Chen, J., Li, F., Zhuang, Q., et al. (2010). A mesenchymal-to-epithelial transition initiates and is required for the nuclear reprogramming of mouse fibroblasts. Cell Stem Cell *7*, 51–63.

Liu, X., Sun, H., Qi, J., Wang, L., He, S., Liu, J., Feng, C., Chen, C., Li, W., Guo, Y., et al. (2013). Sequential introduction of reprogramming factors reveals a time-sensitive requirement for individual factors and a sequential EMT-MET mechanism for optimal reprogramming. Nat. Cell Biol. *15*, 829–838.

Maherali, N., and Hochedlinger, K. (2009). Tgfbeta signal inhibition cooperates in the induction of iPSCs and replaces Sox2 and cMyc. Curr. Biol. *19*, 1718–1723.

Maherali, N., Ahfeldt, T., Rigamonti, A., Utikal, J., Cowan, C., and Hochedlinger, K. (2008). A high-efficiency system for the generation and study of human induced pluripotent stem cells. Cell Stem Cell *3*, 340–345.

Mallanna, S.K., and Rizzino, A. (2010). Emerging roles of microRNAs in the control of embryonic stem cells and the generation of induced pluripotent stem cells. Dev. Biol. *344*, 16–25.

Mani, S.A., Guo, W., Liao, M.J., Eaton, E.N., Ayyanan, A., Zhou, A.Y., Brooks, M., Reinhard, F., Zhang, C.C., Shipitsin, M., et al. (2008). The epithelial-mesenchymal transition generates cells with properties of stem cells. Cell *133*, 704–715.

Marson, A., Foreman, R., Chevalier, B., Bilodeau, S., Kahn, M., Young, R.A., and Jaenisch, R. (2008). Wnt signaling promotes reprogramming of somatic cells to pluripotency. Cell Stem Cell *3*, 132–135.

Melton, C., Judson, R.L., and Blelloch, R. (2010). Opposing microRNA families regulate self-renewal in mouse embryonic stem cells. Nature *463*, 621–626.

Mikkelsen, T.S., Hanna, J., Zhang, X., Ku, M., Wernig, M., Schorderet, P., Bernstein, B.E., Jaenisch, R., Lander, E.S., and Meissner, A. (2008). Dissecting direct reprogramming through integrative genomic analysis. Nature *454*, 49–55.

Park, I.H., Zhao, R., West, J.A., Yabuuchi, A., Huo, H., Ince, T.A., Lerou, P.H., Lensch, M.W., and Daley, G.Q. (2008). Reprogramming of human somatic cells to pluripotency with defined factors. Nature *451*, 141–146.

Rybak, A., Fuchs, H., Smirnova, L., Brandt, C., Pohl, E.E., Nitsch, R., and Wulczyn, F.G. (2008). A feedback loop comprising lin-28 and let-7 controls pre-let-7 maturation during neural stem-cell commitment. Nat. Cell Biol. *10*, 987–993.

Samavarchi-Tehrani, P., Golipour, A., David, L., Sung, H.K., Beyer, T.A., Datti, A., Woltjen, K., Nagy, A., and Wrana, J.L. (2010). Functional genomics reveals a BMP-driven mesenchymal-to-epithelial transition in the initiation of somatic cell reprogramming. Cell Stem Cell *7*, 64–77.

Stadtfeld, M., Maherali, N., Breault, D.T., and Hochedlinger, K. (2008). Defining molecular cornerstones during fibroblast to iPS cell reprogramming in mouse. Cell Stem Cell *2*, 230–240.

Takahashi, K., Tanabe, K., Ohnuki, M., Narita, M., Ichisaka, T., Tomoda, K., and Yamanaka, S. (2007). Induction of pluripotent stem cells from adult human fibroblasts by defined factors. Cell *131*, 861–872.

Vega, S., Morales, A.V., Ocaña, O.H., Valdés, F., Fabregat, I., and Nieto, M.A. (2004). Snail blocks the cell cycle and confers resistance to cell death. Genes Dev. *18*, 1131–1143.

Viswanathan, S.R., Daley, G.Q., and Gregory, R.I. (2008). Selective blockade of microRNA processing by Lin28. Science *320*, 97–100.

Worringer, K.A., Rand, T.A., Hayashi, Y., Sami, S., Takahashi, K., Tanabe, K., Narita, M., Srivastava, D., and Yamanaka, S. (2014). The let-7/LIN-41 pathway regulates reprogramming to human induced pluripotent stem cells by controlling expression of prodifferentiation genes. Cell Stem Cell *14*, 40–52.

Yang, W.H., Lan, H.Y., Huang, C.H., Tai, S.K., Tzeng, C.H., Kao, S.Y., Wu, K.J., Hung, M.C., and Yang, M.H. (2012). RAC1 activation mediates Twist1-induced cancer cell migration. Nat. Cell Biol. *14*, 366–374.

Yu, J., Vodyanik, M.A., Smuga-Otto, K., Antosiewicz-Bourget, J., Frane, J.L., Tian, S., Nie, J., Jonsdottir, G.A., Ruotti, V., Stewart, R., et al. (2007). Induced pluripotent stem cell lines derived from human somatic cells. Science *318*, 1917–1920.

Zhu, H., Shyh-Chang, N., Segrè, A.V., Shinoda, G., Shah, S.P., Einhorn, W.S., Takeuchi, A., Engreitz, J.M., Hagan, J.P., Kharas, M.G., et al.; DIAGRAM Consortium; MAGIC Investigators (2011). The Lin28/let-7 axis regulates glucose metabolism. Cell *147*, 81–94.

Stem Cell Reports, Volume *3*

Supplemental Information

The Epithelial-Mesenchymal Transition Factor SNAI1 Paradoxically Enhances Reprogramming

Juli J. Unternaehrer, Rui Zhao, Kitai Kim, Marcella Cesana, John T. Powers, Sutheera Ratanasirintrawoot, Tamer Onder, Tsukasa Shibue, Robert A. Weinberg, and George Q. Daley

Inventory of Supplemental Information

Supplemental Figures and Legends:

- **Figure S1 (refers to Figure 1)**
- **Figure S2 (refers to Figure 2)**
- **Figure S3 (refers to Figure 3)**
- **Figure S4 (refers to Figure 4)**

Supplemental Table 1.

Supplemental Experimental Procedures

Supplemental References

Figure S1. Gene expression during mouse and human fibroblast and keratinocyte reprogramming.

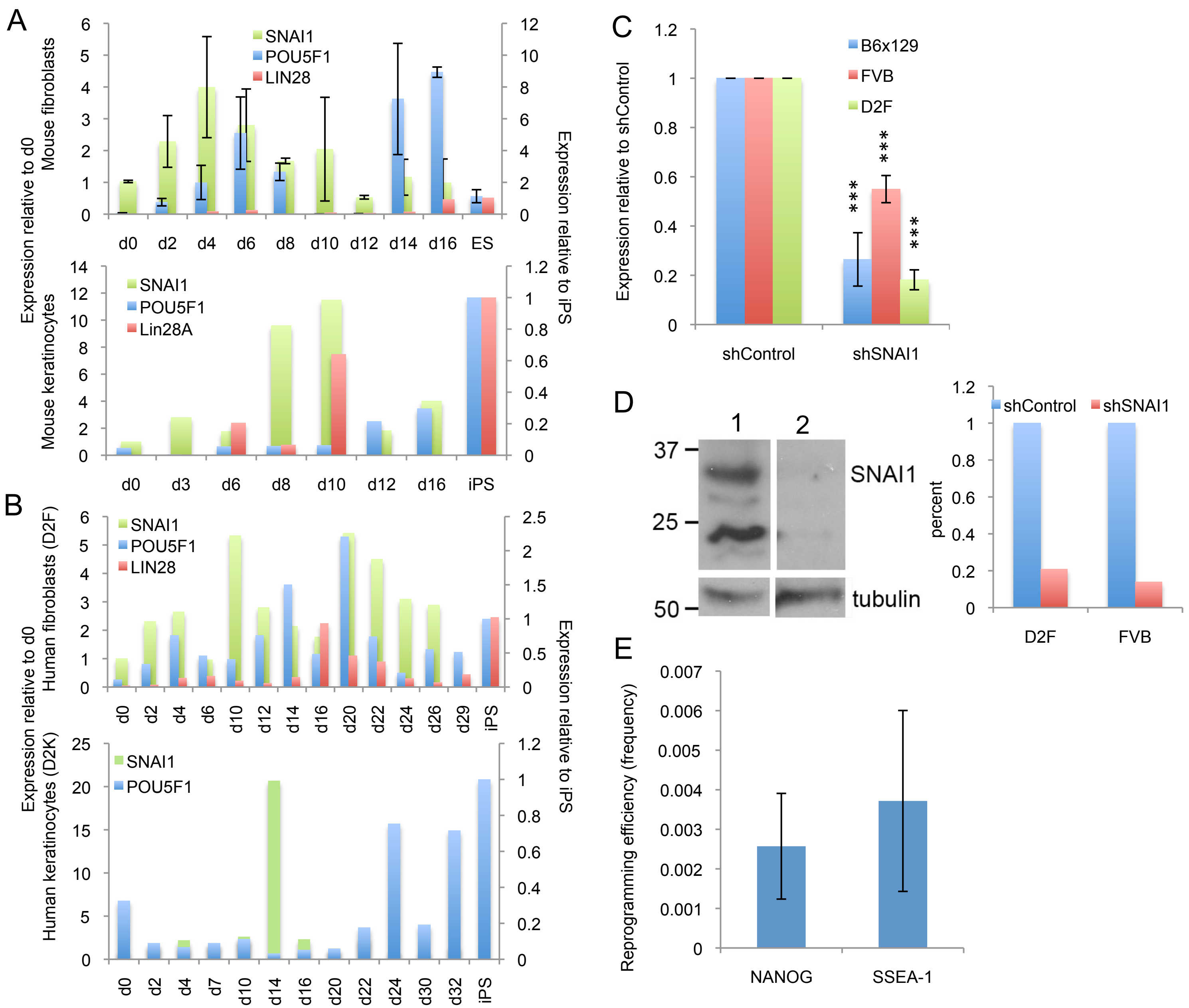

Figure S1. Time course of gene expression during mouse and human fibroblast and keratinocyte reprogramming **(Relates to Figure 1)**.

A. qPCR analyses in mouse cell reprogramming. In mouse fibroblasts (upper panel) and keratinocytes (lower panel) four factors were induced virally or by addition of dox, respectively, and samples were harvested for RNA isolation at indicated time points. After generating cDNA, samples were subjected to qRT-PCR, with normalization to GAPDH. *SNAI1* (green) relative to day 0, endogenous *POU5F1* (blue) and *LIN28* (red) relative to iPS cells. n=3-6 biological replicates; shown are 3 technical replicates for fibroblasts, representative experiment for keratinocytes.

B. qPCR analyses in human cell reprogramming. H1-derived D2F and D2K were reprogrammed by addition of dox, and samples were harvested for RNA isolation at indicated time points. After generating cDNA, samples were subjected to qRT-PCR, with normalization to Actin B. *SNAI1* (green) relative to day 0, endogenous *POU5F1* (blue) and *LIN28* (red) relative to iPS cells. n=3-4 biological replicates; representative experiment shown.

C. qPCR analyses to characterize *SNAI1* knockdown lines. After at least 7d of selection of puromycin-resistant cells, RNA was isolated and cDNA generated, and levels of expression analyzed by qRT-PCR as shown for *SNAI1*, with individual lines for each of three hairpins. Shown is average of three hairpins. n=4-6 biological replicates. ***, $p<0.001$.

D. SNAI1 expression (upper blot) in D2F expressing a control shRNA (lane 1) or shSNAI1 (lane 2) was determined by immunoblotting; lower band is a degradation product of SNAI1 (Zhou et al., 2004). Lower blot, anti-tubulin loading control. Graph at right shows knockdown efficiency in D2F and FVB relative to shControl, normalized to loading control.

E. Colony quantification by labeling with anti-SSEA-1 and anti-NANOG after fixation at day 21 of reprogramming; n=6 technical (NANOG), 2 biological with 6 technical (SSEA-1) replicates. $p>0.2$.

Figure S2. SNAI1 overexpression in human and mouse fibroblasts and keratinocytes.

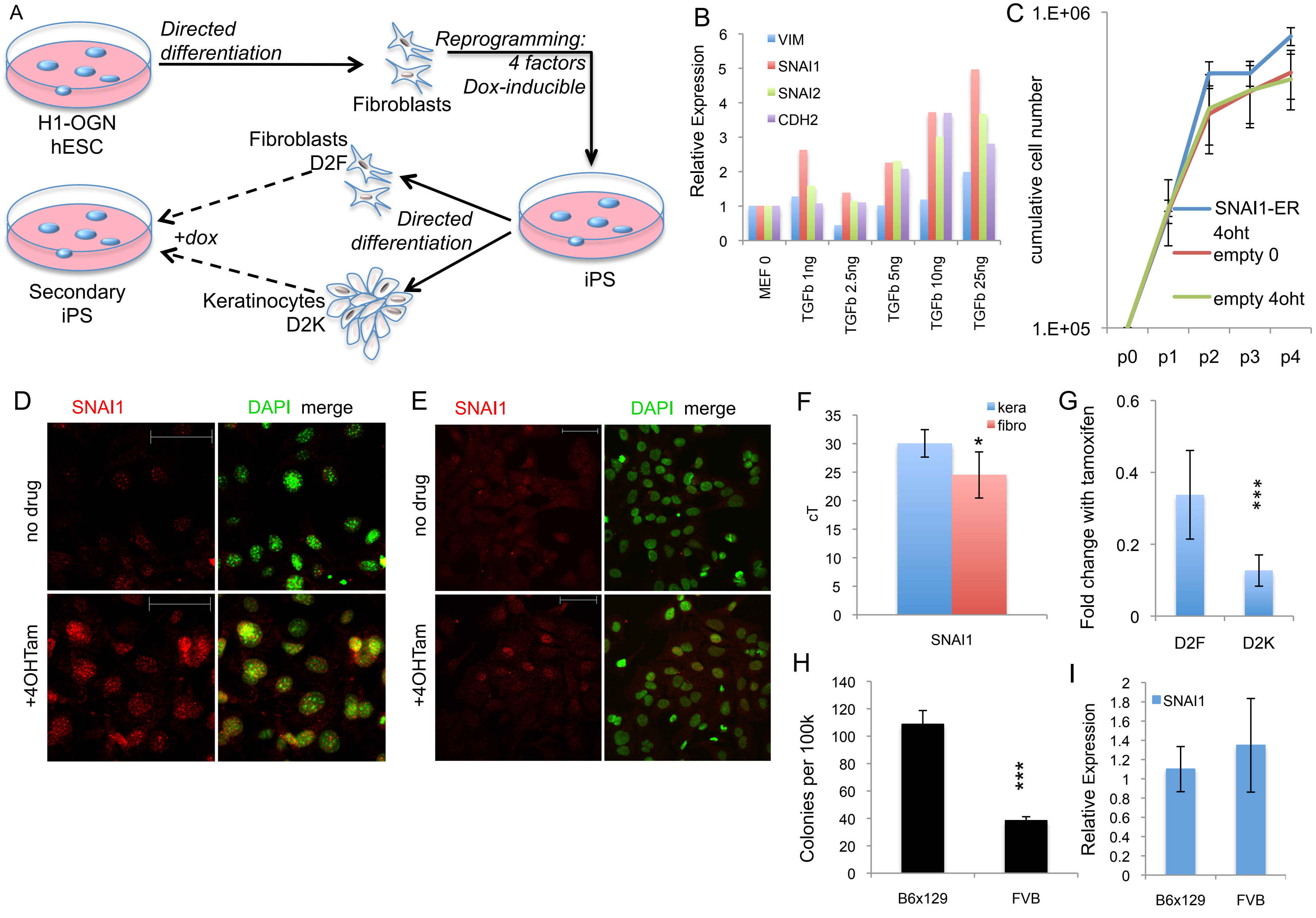

Figure S2. EMT factor overexpression in human and mouse fibroblasts and keratinocytes **(Relates to Figure 2)**.

A. H1 OGN hESCs were differentiated to fibroblasts (Park et al., 2008), followed by transduction with inducible OKSM and rtTA. Doxycycline treatment gave rise to iPS colonies which were picked and expanded. These were in turn differentiated to fibroblasts (D2F) and keratinocytes (D2K), which could then be reprogrammed by addition of Dox, giving rise to secondary iPS cells.

B. Mouse fibroblasts were treated with increasing concentrations of TGF-beta for two days, and expression of EMT factors was determined by qPCR.

C. Proliferation of fibroblasts overexpressing SNAI1-ER or empty vector, +/- tamoxifen. Cells were passaged every three days. n=3 technical replicates.

D,E. Overexpression of SNAI1 in D2F (E) by retroviral transduction and blasticidin selection, then tamoxifen treatment for 12 days, resulted in cells with nuclear-enriched SNAI1 (lower panels). Scale bar, 50um.

F. Expression of SNAI1 in keratinocytes and fibroblasts, strain B6x129. RNA from starting cell populations was processed for qPCR; shown are cT values. n=4, 3 biological replicates. *, $p<0.05$.

G. Effect of tamoxifen on human reprogramming efficiency. D2F and D2K cells bearing an empty vector plasmid, pWZL-GFP, were treated or not with 20nM tamoxifen prior to reprogramming. n=6-8, 3-4 biological replicates. ***, $p<0.001$.

H. Fibroblasts from strains B6x129 and FVB were retrovirally reprogrammed and colonies counted on day 21. n=3 biological replicates. ***, $p<0.001$.

I. SNAI1 expression in starting fibroblasts from strains B6x129 and FVB was determined by qPCR; n=3-5 biological replicates.

Figure S3. Endogenous SNAI1 expression during reprogramming

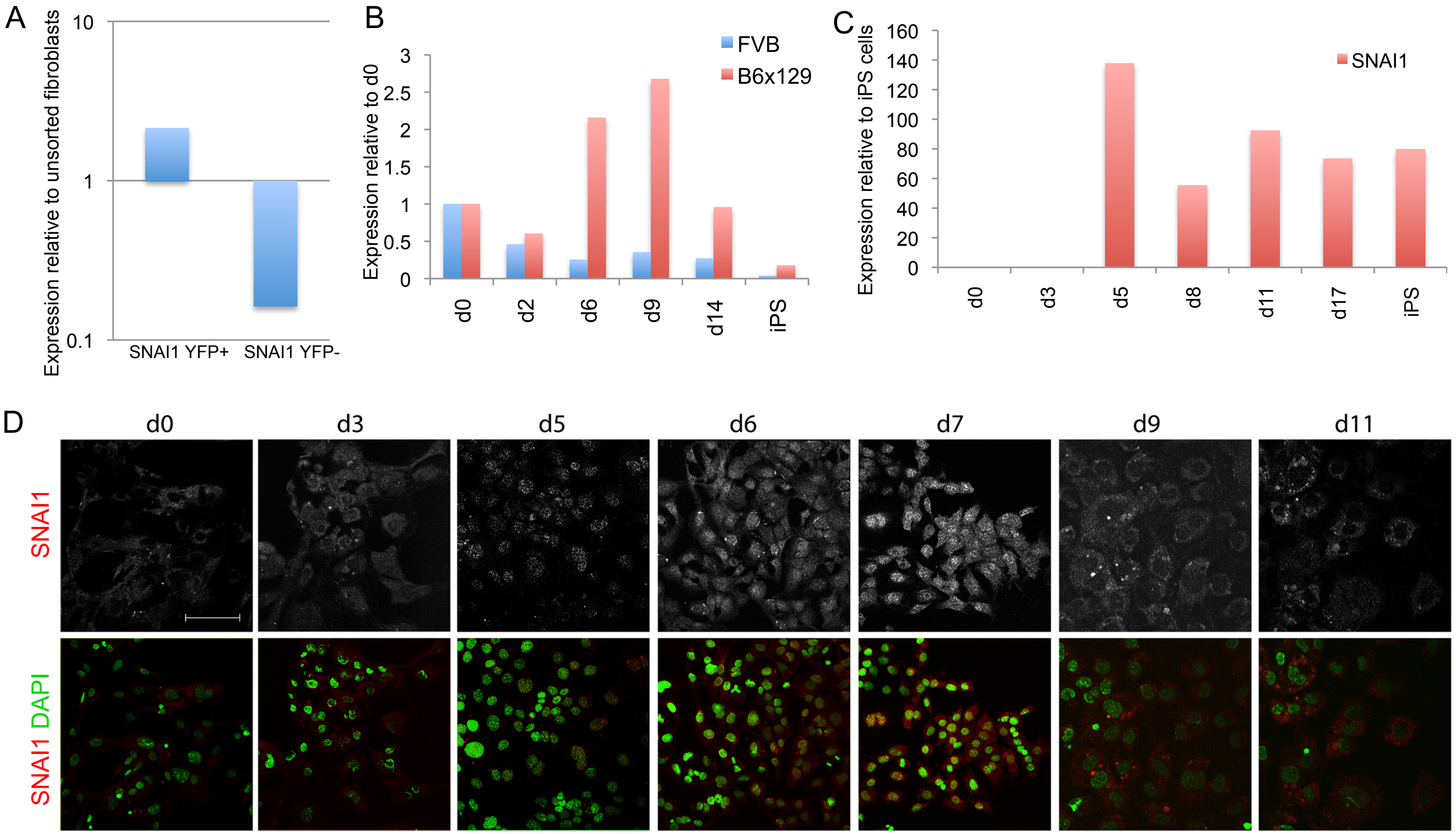

E

Reprogramming efficiency by cell type	Fibroblast	Keratinocyte	Splenocytes
From iOSKM/rtTA mice	0.3%	0.02%	0.01%

Figure S3. Endogenous *SNAI1* expression during reprogramming **(Relates to Figure 3)**.
A. Validation of knock-in reporters. Early passage MEFs from *SNAI1*-YFP knockin mice were sorted for YFP positive and negative fractions. qRT-PCR of resulting cells is shown for *SNAI1*, normalized to unsorted fibroblasts. n=2 biological replicates.
B. Time course of *SNAI1* expression during mouse reprogramming in FVB and B6x129 strains. MEFs from mouse strain B6x129 or FVB were retrovirally reprogrammed, RNA isolated at several timepoints, and qPCR for *SNAI1* performed. n=1.
C. Time course of gene expression during mouse peripheral blood reprogramming. Peripheral blood from iOSKM mice was reprogrammed by addition of Dox, and samples harvested at intervals were analyzed for *SNAI1* expression. n=1.
D. Endogenous SNAI1 expression during mouse reprogramming. iOSKM fibroblasts were reprogrammed by addition of Dox, and labeled by immunofluorescence for SNAI1 at the time points indicated.
E. Reprogramming efficiency (%) from inducible mice from three tissues. N=2-5, 1-3 biological replicates.

Figure S4. Let-7 expression during reprogramming.

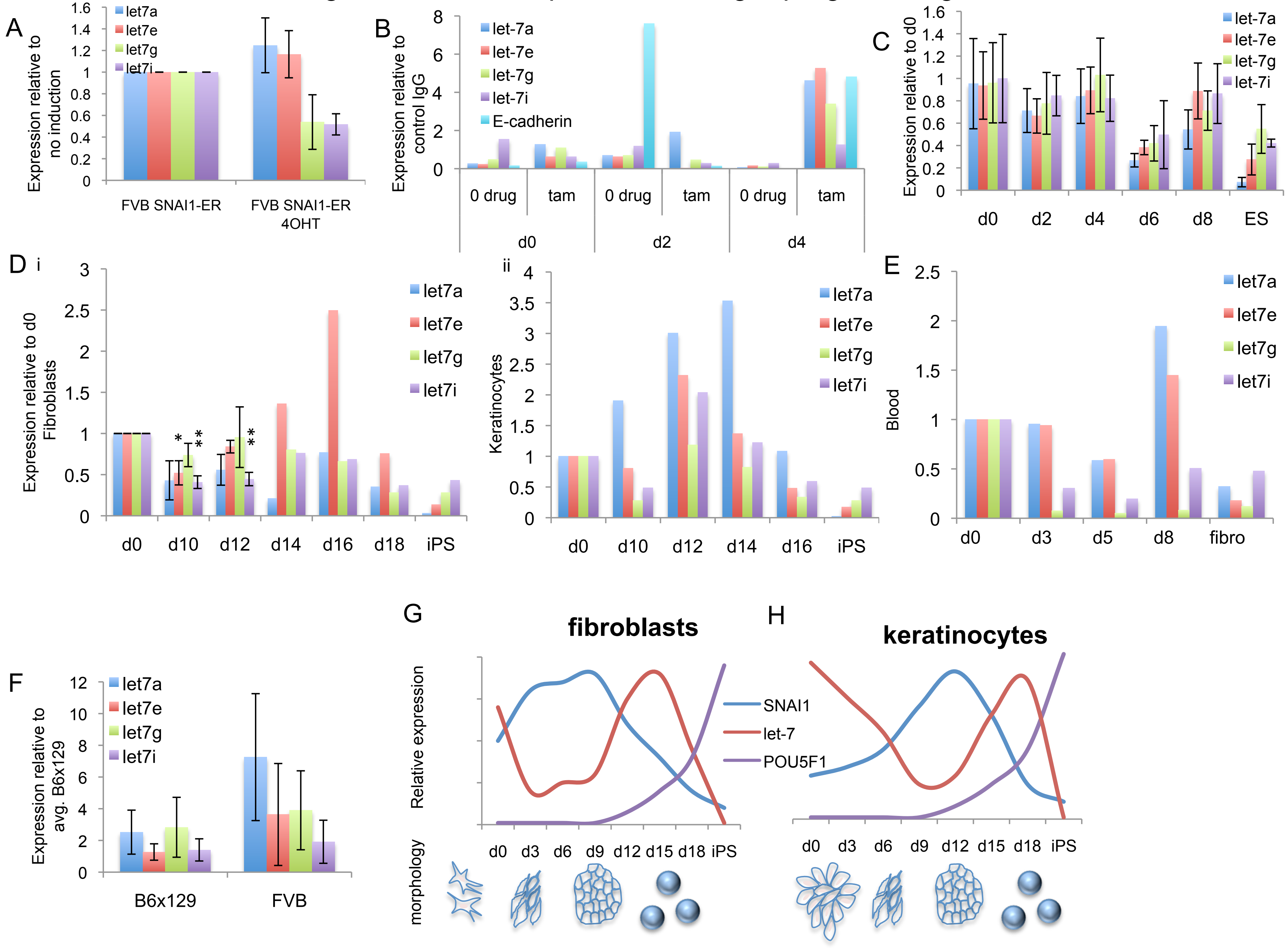

Figure S4. Let-7 expression during reprogramming or SNAI1 manipulation (Relates to Figure 4).

A. Let-7 expression upon SNAI1 overexpression after 10 days of tamoxifen treatment in FVB fibroblasts (n=4, 3 biological replicates) analyzed by TaqMan qPCR.

B. FVB Fibroblasts expressing SNAI1-ER (see figure 2)+/- tamoxifen were reprogrammed and samples collected on d0,2,and 4. ChIP was performed using anti-SNAI1 and binding to let-7 promoters was tested by qPCR. Normalization was to control IgG.

C. Let-7 expression in samples from Figure S1A fibroblasts; n=3 technical replicates.

D. Let-7 expression during the late stages of reprogramming. cDNA prepared from RNA isolated from samples on days 10, 12, 14, 16, 18 of fibroblast (i, n=3-5 biological replicates), or keratinocyte (ii, n=1) reprogramming was analyzed by TaqMan qPCR for let-7 family members; normalization is to day 0.

E. Let-7 expression in peripheral blood reprogramming. RNA samples from days 0, 3, 5, and 8 after dox addition to peripheral blood was analyzed by TaqMan qPCR for let-7 family members; n=1. Normalization is to d0 cells.

F. Let-7 expression in mouse fibroblasts from different mouse strains. RNA from untreated fibroblasts was analyzed for levels of let-7 family members by TaqMan qPCR. Normalization is to the average of the B6x129 samples. n=4 (FVB), 3 (B6x129) (3 biological replicates).

G. In fibroblasts, expression of *SNAI1* increases (blue), and let-7 decreases (red) early in reprogramming. Thereafter, pluripotency factors such as OCT4/*POU5F1* (purple) increase.

H. In keratinocytes, a similar pattern occurs, with slower kinetics.

Table 1. Primers used for qRT-PCR

Gene	Forward	Reverse	Species
Actin	TGA AGT GTG ACG TGG ACA TC	GGA GGA GCA ATG ATC TTG AT	human
GAPDH	ACC ACA GTC CAT GCC ATC AC	TCC ACC ACC CTG TTG CTG TA	mouse
POU5F1	CCT CAC TTC ACT GCA CTG TA	CAG GTT TTC TTT CCC TAG CT	human
	TCT TTC CAC CAG GCC CCC GGC TC	TGC GGG CGG ACA TGG GGA GAT CC	mouse
Snai1	CAC TAT GCC GCG CTC TTT C	GGT CGT AGG GCT GCT GGA A	human
	TCT GAA GAT GCA CAT CCG AAG CCA	AGA CTC TTG GTG CTT GTG GAG CAA	mouse
Snai2	ACT ACA GCG AAC TGG ACA CAC ACA	ACT ACA GCG AAC TGG ACA CAC ACA	mouse
Vimentin	TCT ACG AGG AGG AGA TGC GG	GGT CAA GAC GTG CCA GAG AC	mouse

Supplementary Experimental Procedures:

Mice and Cells: All mouse studies were approved by the Boston Children's Hospital IACUC, and were done in accordance with institutional and national standards and regulations. Mice used in these studies were obtained from the Weinberg lab (*SNAI1*-YFP knockin, mixed Black6/129 background), Jaenisch lab (iOSKM/rtTA, Black6/129 background (Carey et al., 2010), now available from Jackson Labs strain 011004). MEFs were isolated from e14.5 embryos (B6x129, iOSKM, *SNAI1*-YFP, iLet-7s (Zhu et al., 2011), or chimeric mice), or purchased from Chemicon (FVB and B6). iLet-7s mice overexpress Let-7g upon doxycycline treatment. Mouse keratinocytes were isolated from neonatal mice by overnight 5mg/ml Dispase (Stem Cell Technologies) digestion of skin followed by TrypLE select (invitrogen) incubation of epidermis, and were cultured in CnT-07 medium (Cell-N-Tec, ZenBio), and reprogrammed at first passage. Tail tip fibroblasts were cultured from pups or adult mice and used at low passage number. Mouse peripheral blood from iOSKM/rtTA mice (Carey et al., 2010) was obtained by retroorbital bleeding and isolation of buffy coats after settling over 1% dextran and hypotonic red blood cell lysis, and culturing in mouse ESC media with addition of mSCF (50ng/ml), hTPO (20ng/ml), mIL3, mIL6 and Flt3 ligand (10ng/ml). Chimeric mice were created by injection of NP-iPS cells harboring integrated proviruses carrying the four reprogramming factors under doxycycline control (B6x129 background) into blastocysts, which were transferred into pseudopregnant females. Fibroblasts were cultured in DMEM containing 10% FCS, 2mM L-glutamine, 1x penicillin/streptomycin (from 100x stock, Invitrogen), and 100uM 2-mercaptoethanol. Mouse ESC or iPS cell cultures

were cultured in similar media except with 15% FCS, 0.1mM nonessential amino acids (Invitrogen), and 1000U/ml ESGRO (Millipore). Human ESC or iPS cell cultures were grown with media containing DMEM/F12 with 20% knockout serum replacement, 0.1mM non-essential amino acids, 1x penicillin/streptomycin (from 100x stock, Invitrogen), 1mM L-glutamine, 50uM 2-mercaptoethanol, and 10ng/ml bFGF (Invitrogen).

Generation of reporter mouse strain: Reporter mouse strain for the expression of SNAI1 (encoding Snail), *SNAI1*-YFP, was constructed by the following procedure. A gene cassette encoding internal ribosomal entry sequence (IRES), Venus – a mutant of yellow fluorescent protein (YFP; Nagai et al. 2002, Nat Biotechnol 20, 87), and SV40 polyadenylation (polyA) sequence, together with a floxed-neor cassette, were inserted between the termination codon and the endogenous polyA sequence of each locus, doing so by the homologous recombination in v6.5 ES cells (C57BL/6 x 129S4/SvJae Hybrid). The neomycin-resistant clones of v6.5 cells were screened by PCR and Southern blotting. Following the generation of knock-in mice, floxed-neor cassette was removed from these mice by crossing them with CMV-Cre mice.

Genotyping: Mouse genotyping was done by PCR analysis of tail DNA (primers available upon request) using DNeasy kits (Qiagen), or by Transnetyx assay.

Fluorescence microscopy: Reprogrammed cells were plated on coverslips on d0 or d3 (mouse) or d5 (human) and cultured for an additional 1-5 days, then fixed in ice cold methanol followed by antibody labeling as follows: 30min incubation in permeabilization solution (PS)+ (0.05% saponin, 10mM HEPES, 10mM glycine, 10% goat serum (Invitrogen) in DMEM), 30min incubation in primary antibody diluted in PS+, 30min incubation in secondary antibody in PS+, 5min incubation in 300nM DAPI (Invitrogen), and mounting on slides with ProLong antifade reagent (Invitrogen). Cells were visualized on a Zeiss 510 confocal microscope equipped with a 40x objective.

Antibodies: Anti-SNAI1 (H-130, for immunofluorescence) was from Santa Cruz, or ab85931 from Abcam (for Western blot). Secondary antibodies were goat anti-mouse, -rat, or -rabbit Alexa Fluor 488, 568, and 647 dyes, or for Western blot, goat anti-rabbit HRP (Invitrogen).

Antibody labeling of reprogramming plates: After fixation in 4% PFA, plates were labeled with biotinylated primary antibodies and streptavidin-horseradish peroxidase (Biolegend), and visualized by diaminobenzidine reaction (Vector labs).

Reprogramming: Viral-mediated mouse reprogramming was by spinoculation of 10e5 cells per well of 6 well plate 12-24h after plating with pMX-Oct4, Sox2, Klf4, and pEYK-c-Myc viral supernatants with protamine sulfate (Sigma-Aldrich) at 6ug/ml, 2500rpm for 90min at 25°C. Individual wells of bulk cultures were harvested for assays. Colonies were counted on d21-35 (mouse) or d28-42 (human) by the following criteria: 1) colony morphology (smooth refractive well-defined borders), 2) SSEA-1 (mouse) or Tra1-60 (human, both from eBioscience) labeling. Efficiency calculations were made by counting colonies (ImageJ) of all conditions fixed on the same day according to the following formula: colonies per 100K cells = (100000 x colonies counted)/number of cells replated on d3 (mouse) or d5 (human). Non-normalized colony counts (Figure 3B) compared raw numbers of colonies obtained from an equal number of starting cells. For human reprogramming, tamoxifen alone decreased the efficiency of reprogramming (Figure S2F); efficiencies included a tamoxifen correction factor as follows: efficiency*(control efficiency without tamoxifen/control efficiency with tamoxifen).

Lentiviral knockdown: Lentivirus containing knockdown constructs was produced by co-transfection of 293T cells with lentiviral constructs, gag/pol, and VSV-G using Fugene6 (Roche). Supernatants 48 and 72 hours after transfection were harvested, filtered and stored at -80oC. Target cells were transduced by spinoculation of unconcentrated supernatants as for reprogramming. Stable lines were selected for at least 1 week. pLK0.1-puromycin (puro)-based constructs against SNAI1 were obtained from Sigma (SHDNA-NM_011427, SHDNA-NM_011415). Lentivirus containing KD constructs was

produced as described (Onder et al., 2012) and transduced by spinoculation as for reprogramming. Stable cell lines were selected by addition of Puro at 1ug/ml on day 2 following transduction, continuing for at least 1 week.

Retroviral overexpression: pWZL-based vectors expressing Twist- or SNAI1-ER, or a control empty vector (Mani et al., 2008) were used to generate virus, and transduced by spinoculation as above. Cells were induced for at least 10 days by addition of 4-OH-tamoxifen (Sigma-Aldrich) at 20nM, followed by reprogramming. Lines were selected starting on day 2 after transduction using 5 ug/ml Blasticidin and continuing for at least one week.

Quantitative real-time PCR (qRT-PCR): RNA was isolated using Trizol or RNeasy mini- or micro kits (Qiagen), and cDNA was generated from 1ug RNA using the Superscript III first strand synthesis kit (Invitrogen). Results were normalized by comparison to GAPDH (mouse) or Actin B (human). qRT-PCR was performed using SYBR green master mix (Stratagene) on a Stratagene Mx3000P. Primer sequences are listed in Supplementary Table 1 online. microRNA was isolated using Trizol (Invitrogen) or miRNeasy (Qiagen), cDNA was generated from 100ng RNA using TaqMan microRNA reverse transcription kit (Applied Biosystems), and levels of mature miRNA were detected using commercially available TaqMan

probes (Applied Biosystems) per manufacturer's instructions with sno142 RNA as internal standards for normalization. miR qPCR was done as described (Viswanathan et al., 2008).

Chromatin immunoprecipitation assays: ChIP analyses were carried out on chromatin extracts according to manufacturer's specifications (MAGnify ChIP – Invitrogen) with anti-SNAI1 from Abcam, ab85931 and from Cell Signaling Technology, L70G2. Data are represented as fold enrichment with respect to control antibody (IgG), normalized to background signal of specific antibody over a negative genomic region. Let-7a-2, let-7g, let-7e, and let-i were analyzed, with oligos designed from a region comprising 2kb upstream of the stem-loop of the first member of the cluster. Primer pairs were tested for amplification efficiency by generating a standard curve over five dilutions of input sample. Data are representative of three independent experiments. Primer sequences are available upon request.

Statistics: Statistical analysis was performed using the Student's two-tailed t-test. *, $p<0.05$; **, $p<0.01$; ***, $p<0.001$. Error bars indicate SEM.

Supplemental references.

Carey, B.W., Markoulaki, S., Beard, C., Hanna, J., and Jaenisch, R. (2010). Single-gene transgenic mouse strains for reprogramming adult somatic cells. Nat Methods *7*, 56-59.

Mani, S.A., Guo, W., Liao, M.J., Eaton, E.N., Ayyanan, A., Zhou, A.Y., Brooks, M., Reinhard, F., Zhang, C.C., Shipitsin, M.*, et al.* (2008). The epithelial-mesenchymal transition generates cells with properties of stem cells. Cell *133*, 704-715.

Onder, T.T., Kara, N., Cherry, A., Sinha, A.U., Zhu, N., Bernt, K.M., Cahan, P., Marcarci, B.O., Unternaehrer, J., Gupta, P.B.*, et al.* (2012). Chromatin-modifying enzymes as modulators of reprogramming. Nature *483*, 598-602.

Viswanathan, S.R., Daley, G.Q., and Gregory, R.I. (2008). Selective blockade of microRNA processing by Lin28. Science *320*, 97-100.

Zhou, B.P., Deng, J., Xia, W., Xu, J., Li, Y.M., Gunduz, M., and Hung, M.C. (2004). Dual regulation of Snail by GSK-3beta-mediated phosphorylation in control of epithelial-mesenchymal transition. Nat Cell Biol *6*, 931-940.

Zhu, H., Shyh-Chang, N., Segre, A.V., Shinoda, G., Shah, S.P., Einhorn, W.S., Takeuchi, A., Engreitz, J.M., Hagan, J.P., Kharas, M.G.*, et al.* (2011). The Lin28/let-7 axis regulates glucose metabolism. Cell *147*, 81-94.
